# A novel and cost-effective method for high-throughput 3D culturing and rhythmic assessment of hiPSC-derived cardiomyocytes using retroreflective Janus microparticles

**DOI:** 10.1186/s40824-023-00416-4

**Published:** 2023-08-16

**Authors:** Huyen T. M. Pham, Duc Long Nguyen, Hyo-Sop Kim, Eun Kyeong Yang, Jae-Ho Kim, Hyun C. Yoon, Hyun-Ji Park

**Affiliations:** Department of Molecular Science and Technology, Ajou University, Suwon, 16499 South Korea

**Keywords:** 3D cell culture platform, hiPSC-CM, Retroreflective Janus microparticle (RJP), Cardiac rhythm assessment

## Abstract

**Background:**

Human induced pluripotent stem cell-derived cardiomyocytes (hiPSC-CMs) gain attention as a potent cell source in regenerative medicine and drug discovery. With the necessity of the demands for experimental models to create a more physiologically relevant model of the heart in vitro we herein investigate a 3D culturing platform and a method for assessing rhythm in hiPSC-CMs.

**Methods:**

The 3D cell culture PAMCELL™ plate is designed to enable cells to attach exclusively to adhesive patterned areas. These cell adhesive zones, named as micro-patterned pads, feature micron silica beads that are surface-modified with the well-known arginyl-glycyl-aspartic acid (RGD) peptide. RGD binding to the surface of hiPSC-CMs facilitates cell–cell attachment and the formation of uniform-size spheroids, which is controlled by the diameter of the micro-patterned pads. The assessment and evaluation of 3D hiPSC-CMs beating pattern are carried out using reflective properties of retroreflective Janus micro-particle (RJP). These RJPs are modified with an antibody targeting the gap junction protein found on the surface of hiPSC-CM spheroids. The signal assessment system comprises a camera attached to an optical microscope and a white light source.

**Results:**

The 3D PAMCELL™ R100 culture plate efficiently generate approximately 350 uniform-sized hiPSC-CM spheroids in each well of a 96-well plate and supported a 20-day culture. Analysis of genes and protein expression levels reveal that iPSC-CM spheroids grown on PAMCELL™ R100 retain cardiac stem cell characteristics and functions, outperforming traditional 2D culture platform. Additionally, the RJPs enable monitoring and evaluation of in vitro beating properties of cardiomyocytes without using complex monitoring setup. The system demonstrates its capability to identify alteration in the rhythmic activity of cardiac cells when exposed to ion channel blockers, nifedipine and E4031.

**Conclusions:**

The integration of the 3D culture method and RJPs in this study establishes a platform for evaluating the rhythmic properties of 3D hiPSC-CMs. This approach holds significant potential for identifying arrhythmias or other cardiac abnormalities, ultimately contributing to the development of more effective therapies for heart diseases.

**Graphical Abstract:**

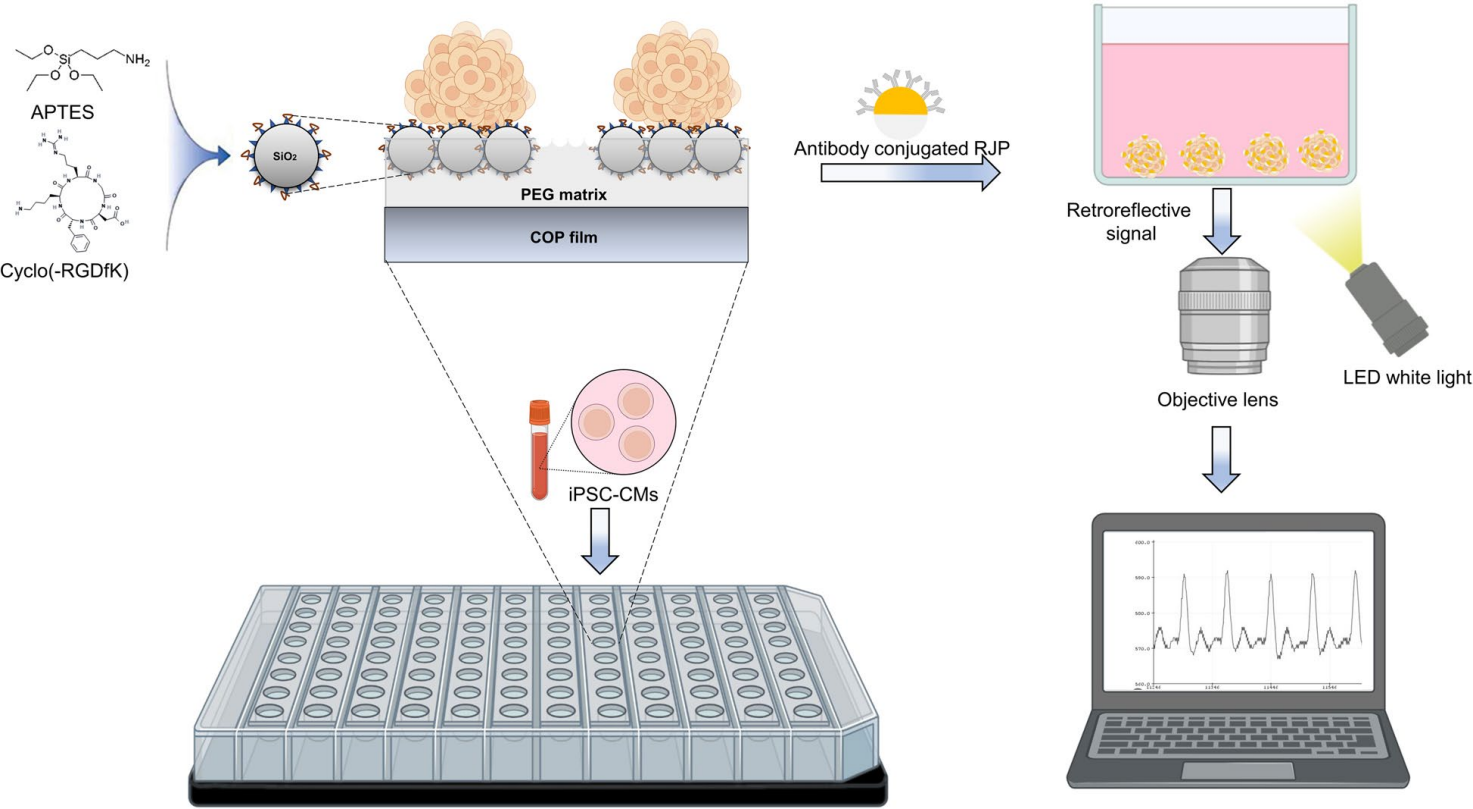

**Supplementary Information:**

The online version contains supplementary material available at 10.1186/s40824-023-00416-4.

## Background

Cardiovascular diseases (CVD) accounted for over 19 million deaths worldwide in 2022, with the majority of these fatalities occurring in low- and middle-income countries [1, 2]. The growing prevalence of CVD has led to an urgent need for a deeper understanding of the underlying mechanisms and the development of novel diagnostic and therapeutic approaches. Over the past few decades, CVD research has broadened to encompass various areas, including genomics and genetic engineering [3, 4], biomarker discovery [5–7], and molecular and cellular mechanism studies [8–10] to pursue improved therapeutics strategies [11–13]. To better understand of CVD progression and develop improved therapeutic strategies, it is critical to develop suitable in vitro models using stable cell lines with an unlimited cell supply. In this context, cardiomyocytes (CMs) derived from human induced pluripotent stem cells (hiPSC-CMs) have emerged as ideal candidates to meet these requirements.

In recent years, three-dimensional (3D) in vitro models have gained attention in CVD research, as they accurately simulate the in vivo microenvironment compared to traditional two-dimensional (2D) models [14]. Indeed, 3D models can better replicate the structural and mechanical properties of the heart, facilitating the study of cardiac disease progression and drug testing in a more physiologically relevant environment [15–17]. However, conventional spheroid culture platforms present technical challenges for maintaining spheroids and their downstream applications, such as difficulty in tracking specific cells within spheroids during live imaging and the loss of spheroids during media change. These challenges highlight the need for an advanced culture platform to address these issues and enable broader use of 3D models in CVD research.

One key aspect that benefits from adhesion culture of spheroids is live cell tracking in 3D environment. In particular, for iPSC-CM spheroids, the extent and delay of beating, as well as abnormal beating patterns, can provide crucial diagnostic and prognostic information as in arrhythmia or tachycardia [18–20]. However, with non-adhesive culture platforms, accurately tracking the movement of individual cells or the entire spheroid can be challenging due to the displacement caused by the beating of the CMs. Moreover, current techniques to observe cell beating primarily rely on fluorescent Ca^2+^ indicators, such as Fura-2 and Indo-1, which enable the observation of cell beating through fluorescent microscopy. However, these methods are devised to indicate Ca^2+^ propagation, making them indirect measures of cell beating, as beating is a consequence of Ca^2+^ propagation in CMs. These methods also have limitations including high costs, potential for photobleaching, and cell damage from blue light irradiation [21, 22]. Thus, there is a need for alternative approaches that directly measure cell beating. Retroreflective Janus micro-particles (RJPs) can be utilized to analyze beating of hiPSC-CMs, as they non-invasively label cells with minimal cytotoxicity and can be observed through optical microscopy. Specifically, RJPs can reflect polychromatic white light and other visible light, allowing for easy detection of their angular displacement with a conventional microscope and a white LED optical component [23–31].

Together, we present a high-throughput spheroid culture platform to measure the beat rate of hiPSC-CM spheroids. To achieve this, we functionalized surface of micron silica patterned PAMCELL™ R100 cell culture platform with Arginyl-glycyl-aspartic acid (RGD). RGD is a tripeptide sequence that is commonly found in extracellular matrix proteins such as fibronectin, vitronectin, and collagen [32]. The RGD peptides play a crucial role in cell adhesion and migration through its interaction with integrin receptors [33, 34]. Additionally, positive charges of the peptides significantly enhance the nonspecific cell adhesion [35]. Therefore, immobilizing RGD peptides on surfaces of biomaterials is one of the approaches in 3D culturing and tissue engineering [36, 37]. Thus, the PAMCELL™ R100 plate is prepared by arraying RGD-functionalized silica particles into the specific patterns on poly (lactic-co-glycolic acid) (PLGA), Polyethylene terephthalate (PET), or Cyclo Olefin Polymer (COP) film (ANK Corporation, Suwon, South Korea). The PAMCELL™ R100 plate has been shown to be effective for culturing as 3D spheroids with various types of cells, including stem cells [38, 39].

Here, we demonstrated a novel 3D cell culture platform, the PAMCELL™ R100 plate, capable of generating hiPSC-CM spheroids that maintain high cell viability for prolonged culture periods. Additionally, we employed RJPs as a means to detect hiPSC-CM spheroid contractions. This innovative approach offers a stable, high-throughput, cost-effective, and user-friendly solution for studying CM spheroids.

## Materials and methods

### Preparation of the hiPSC-CM spheroid culture plate

**Fig. 1:**
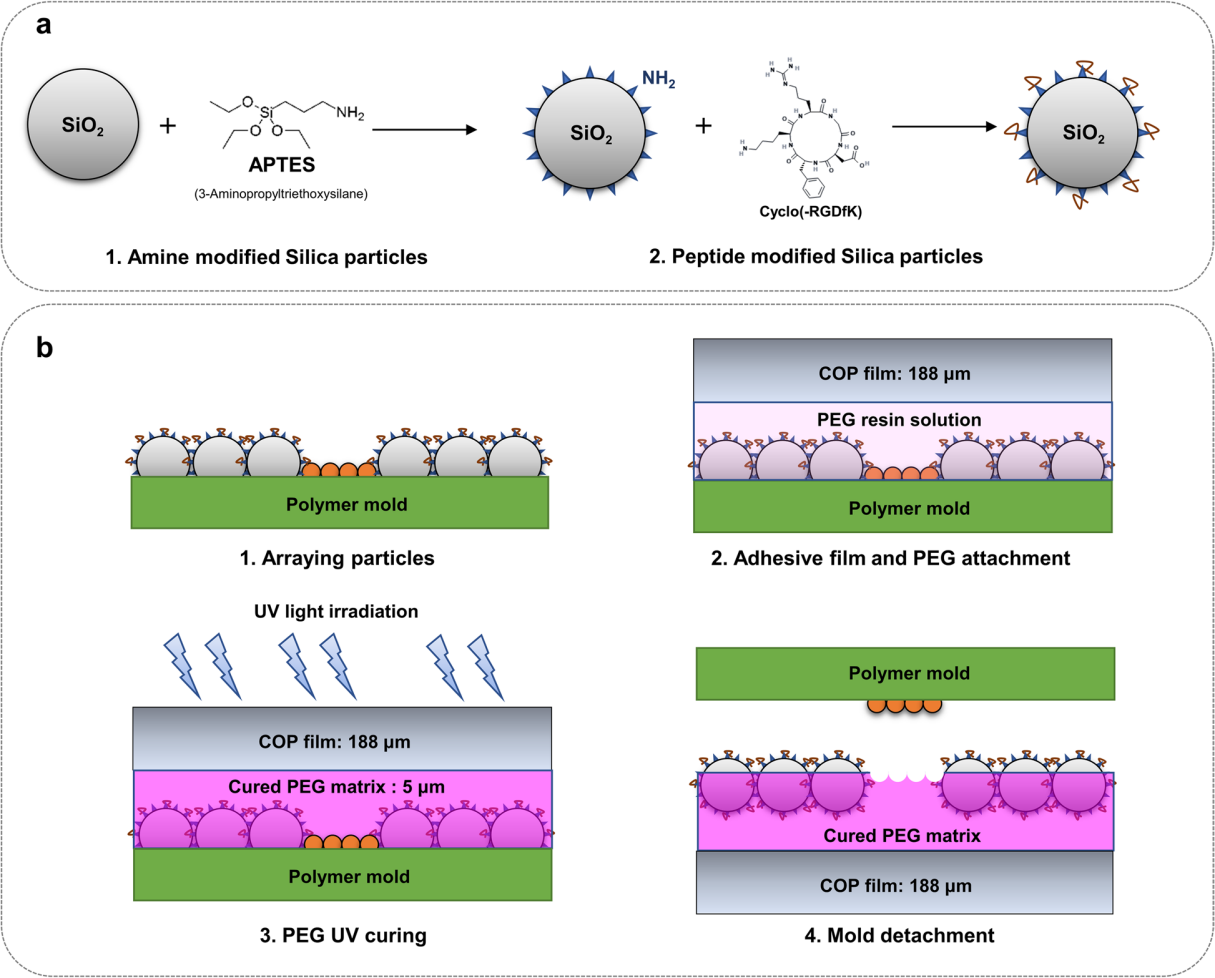
Schematic cross-sectional diagram of PAMCELL™ R100 plate preparation. (**a**) Conjugation of Amide and RGD on the surface of Silica particles; (**b**) Particle array and pattern

The PAMCELL™ R100 culture plates were developed and characterized by the ANK Corporation (Suwon, South Korea), as described in Fig. 1. Briefly, 1 µm silica particles (Sakai Chemical Industry Co., Ltd., Tokyo, Japan) were functionalized with cyclic-RGD (Arg-Gly-Asp) peptides (Peptron, Daejeon, South Korea) by ((3-Aminopropyl)triethoxysilane (APTES; Sigma–Aldrich, St. Louis, MO, USA) (Fig. 1a). The cyclic-RGD functionalized silica particles were then densely-packed arrayed in a monolayer on a non-fouling polymer, polyethylene glycol (PEG; Sigma–Aldrich) and then PEG was cured by UV (Fig. 1b).

Fluorescein isothiocyanate (FITC) conjugated RGD peptide (FITC-RGD; Peptron) was employed to confirm the successful binding of RGD peptide to silica particles. Briefly, conjugation of FITC-RGD and silica particle was measured by FP-8200 Spectrofluorometer (JASCO International Co. Ltd., Tokyo, Japan) (Supplementary Fig. S1a). After verifying conjugation, FITC-RGD coated silica particles were introduced to the PAMCELL™ R100 plate and allowed to adhere to the adhesive pad region. The green fluorescence from the particles was captured using the Cell3iMager duos 2 CC8300 (SCREEN Holdings Co., Ltd., Kyoto, Japan) (Supplementary Fig. S1b-d). The plate consists of a black polystyrene body and a clear, 188 µm thick, fluorescence-free COP (Cyclic olefin polymer) film substrate (Zeon Corp., Tokyo, Japan). Plates were sterilized with EO gas and packaged in aluminum packs before use in cell culture.

## hiPSC-derived cardiomyocyte culture and maintenance

HiPSC-CMs was obtained from NEXEL Co., Ltd., Seoul, South Korea. For standard two-dimensional monolayer (2D) culture, cryopreserved single-cell suspensions was rapidly thawed and diluted in pre-warmed Cardiosight-S Advanced Plating Medium (CAPM). hiPSC-CMs were seeded into culture dishes coated with Matrigel (Corning Inc., NY, USA) at a 1:100 ratio in ice-cold DMEM/F12 (Gibco™, NY, USA) followed by Nexel Cardiosight-S user guide V2022. The medium was changed to Cardiosight-S Advanced Maintenance Medium (CAMM) after 18 h. Starting on the day after plating the cells, the maintenance media was changed every day. For cardiac spheroid culture, 8 × 10^5^ of viable hiPSC-CMs were diluted in 300 µL CAMM and seeded into 96-well PAMCELL™ R100 coated with iMatrix-511 silk (NIPPI Corp., Kanagawa, Japan) in cell incubator for at least an hour. The media was half change every day. The control hiPSC-CM spheroids were generated using the 24-well AggreWell™ 400 plates (STEMCELL Technologies Inc., Vancouver, Canada) following the manufacturer’s protocol. Briefly, the plate was treated with 500 µL Anti-Adherence Rinsing Solution (STEMCELL Technologies Inc.) per well and then centrifuged at 4,000 rpm for 5 min to remove bubble and prevent cell adhesion. The rinsing solution was then replaced by 1 mL of CAPM and aspirated before seeding cells. 3,600 viable hiPSC-CMs were diluted in 1 mL CAPM and seeded to a well (300 cells per micro-well). The plate was then centrifuged at 100 × *g* for 5 min. Within 24 h after seeding day, media was replaced by CAMM. From day 2, the media was half change every day. Cultures were maintained at 37ºC in a humidified atmosphere containing 5% CO_2_.

### Retroreflective Janus Microparticles (RJP) preparation and treatment

**Fig. 2:**
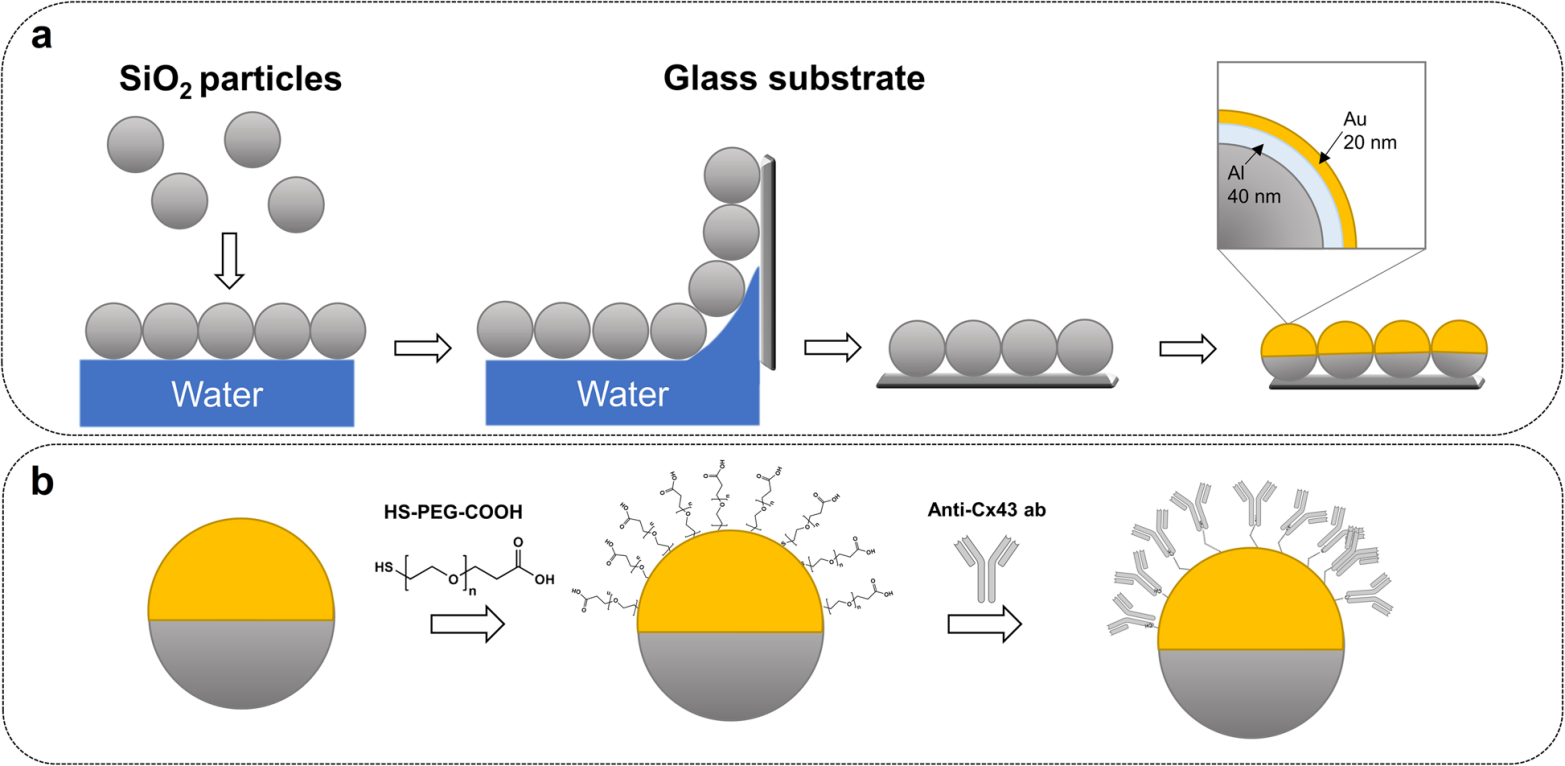
Schematic diagram of RJP preparation. (**a**) bare RJP preparation; (**b**) Conjugation of antibody on the surface of RJP

Uniform silica microparticles with the diameter of 1.5 µm were purchased from Bangs Laboratories, Inc. (ThermoFisher, IN, USA). Prior to the preparation of microparticles, the surfaces of silica microparticles were subjected to surface modification using carbonyldiimidazole (CDI). The surface-modified particles were then dispersed in chloroform, followed by monolayer spreading on the water surface. Subsequently, the aforementioned particles were collected on BK glass using the Langmuir Blodgett methodology [40]. The glass substrates were air-dried and then subjected to electron beam physical vapor deposition to generate a metallic layer (Fig. 2a).

Prior to antibody conjugation, the particles were subjected to a 3-h reaction with 1 mM Thiol polyethylene glycol acid (HS-PEG-COOH) (MW 1,000 Da) (Creative PEGWorks, Durham, NC, USA). Following the washing with deionized water (DW), RJPs were treated with an *N*-ethyl-*N′*-(3-(dimethylaminopropyl)carbodiimide/*N*-hydroxysuccinimide (EDC/NHS) reaction (final concentration EDC 10 mM/ NHS 10 mM) for 1 h (Fig. 2b). The particles were then washed with 0.5 M 2-(*N*-morpholino)ethanesulfonic acid (MES) buffer at pH 5.5 three times Afterward, RJPs were treated with 100 μg/mL of the anti-connexin 43 antibodies for 1 h at 4℃. The particles were then washed with 50 mM Phosphate-Buffered Saline (PBS) in 0.05% Tween (PBST) and treated with 20 mM ethanolamine for 1 h. RJPs were blocked by incubating the particles in a blocking buffer (0.05% PBST containing 1% bovine serum albumin (BSA)) for 1 h at 4℃. The particles were sonicated and then spun down using a centrifuge with 0.05% PBST. Afterward, the particles were washed with PBST were then stored in the same buffer.

To verify the antibody surface modification, Alexa Fluor™ 488-conjugated anti-rabbit IgG were treated to antibody conjugated RJPs (Ab-conjugated RJPs) and negative control (bare RJPs) for 30 min at room temperature. Samples were then washed and injected to slide glass before taking images by a fluorescent microscope (Leica DMI 4000b, Wetzlar, Germany).

### Cell viability assessment

Live/dead™ Viability/Cytotoxicity Kit, for mammalian cells (Invitrogen, Carlsbad CA, USA) was used to analyze cell viability after 30-min incubation with 2 µM Calcein AM (C-AM) and 4 µM Ethidium homodimer-1 (EthD-1) in cell media. Calcein AM produced an intense uniform green fluorescence in live cells and Ethidium homodimer-1 produced a bright red fluorescence in dead cells. Fluorescent photos were imaged under the Cell3iMager duos 2 CC8300 (SCREEN Holdings Co., Ltd., Kyoto, Japan).

### Ca^2+^ flux measurement

For imaging of the Ca^2+^ transient, the spheroids cultured on PAMCELL™ R100 plate were loaded with 1 μM pluronic F-127 (Invitrogen) and 1 μg Fura-2/AM (Invitrogen) for 30 min in 37℃, 5% CO_2_ incubator. Ca^2+^ transient videos were captured by a confocal microscope (Nikon A1; Nikon, Minato, Japan) with camera at a resolution of 512 × 512 pixels and 24 fps using a 20 × objective and recorded using NIS software (Nikon). The data were then quantified as the background subtracted fluorescence intensity changes (ΔF) normalized to the baseline fluorescence (F0) using the ImageJ software v.1.53 (NIH, Bethesda, USA).

### Flow cytometry analysis

FACS analysis of 3D hiPSC-CMs was carried out on day 8 after culturing as well as 2D controls. The spheroids were dissociated into single cells using Accutase (ThermoFisher Scientific) for 10 min and fixed with a 4% paraformaldehyde solution (PFA) for 20 min. The fixed cells were then permeabilized with 1% Triton-X 100 for 10 min. Samples were treated with FACS blocking buffer (50 mM PBS containing 1% BSA) and then incubated with anti-Cardiac Troponin T antibody (cTnT), anti-calcium voltage-gated channel subunit alpha 1C (cav1.2), anti-Myosin light chain 7 (MYL7), anti-Desmin, and anti-Connexin 43 (Supplementary Table S1) in FACS solution (50 mM PBS containing 1% BSA, 10% fetal bovine serum (FBS)) for 30 min 4℃ after washing. Alexa Fluor™ 488-conjugated anti-rabbit IgG (ThermoFisher) was treated as secondary antibodies for 30 min at 4℃. Cardiomyocytes were then analyzed using NovoCyte Flow Cytometer Systems (Agilent Technologies, Santa Clara, CA, USA).

### Immunocytochemistry

2D cultured hiPSC-CMs were fixed with 4% PFA for 15 min. The fixed cells were then permeabilized with 0.3% Triton-X 100 for 10 min. After washing with PBS, samples were treated with blocking buffer (50 mM PBS containing 1% BSA, 0.1% Triton X-100) for 30 min. The permeable cells were incubated with antibodies against cTnT, cav1.2, MYL7, Desmin, and Connexin 43 (Supplementary Table S1) for 1 h at 4℃. Secondary antibodies were treated for 30 min at 4℃. The nuclei of iPSC-CMs were stained using 4′,6-Diamidino-2-phenylindole dihydrochloride (DAPI, 1:1000 dilution in PBS, ThermoFisher) for 10 min.

hiPSC-CM spheroids cultured on PAMCELL™ R100 plate was followed the same protocol with time adjustment. Fixation, permeability, and blocking time are 1 h, 30 min, and 1 h respectively. Washing steps were repeated three times. Spheroids were noticed to not be exposed to the air for every single step to avoid being dry and de-attach from plates.

In case of the AggreWell™ 400 plate, spheroids were taken out from the wells, transferred to 1.7 ml centrifuge tube, and centrifuged at 2300 × g for 5 min 4 °C. Media was aspirated and replaced by 1 mL 4% PFA. After 30 min of fixation. The supernatant was discarded with careful attention and spheroids were re-suspended with 0.3% Triton-X 100 for 30 min after washing with PBS. The blocking step of permeable cells was done for 1 h after removing Triton-X100 by PBS. The 1st antibodies (Supplementary Table S1) were then treated and incubated overnight at 4℃. Spheroids were incubated with 2nd antibodies for 1 h in RT. The nuclei of the spheroids were stained with DAPI for 20 min. For slide preparation and taking fluorescent images, spheroids were washed, and the supernatant was carefully discarded. 50 µL PBS were added for resuspension and a P200 with the end of the tip cut off was used to pipette the spheroids to slide glass. After 5 min-settlement, the leftover PBS was aspirated without disturbing the spheroids. Faramount Mounting Medium (Agilent Technologies) were treated and a coverslip was placed without creating bubbles. The edges of the cover glass were sealed and cured in the dark for 5 to 10 min. The slide glasses were then stored at 4℃.

Florescent images were taken using a confocal microscope (Nikon A1R-HD25; Nikon).

### Real-time polymerase chain reaction to quantify cardiac mRNA expression

Total mRNA of hiPSC-CMs at day 8 of culturing was extracted with RNA-spin™ Total RNA Extraction Kit (iNtRON Biotechnology, Inc., South Korea) according to the manufacturer’s instructions, and quantified with NanoDrop One (ThermoFisher). RT-PCR was carried out according to manufacturer's instructions using Maxime™ RT PreMix (Random Primer) (iNtRON Biotechnology, Inc.) to generate total cDNA. All PCR reactions run in AriaMx Real-Time PCR (qPCR), using 1 μL cDNA template, 7 μL ultra-distilled water, 10 μL RealMOD green W^2^ 2 × qPCR mix (iNtRON Biotechnology, Inc.), and 1 μL of forward and backward primer each. Information of primers were used was described in the Supplementary Table S2. PCR setting consisted of initial denaturation at 95 °C for 10 min, 40 cycles of denaturation for 20 s at 95 °C, annealing for 40 s at 55 °C, melt at 1 cycle of 95 °C for 10 s, 65 °C for 10 s, and 95 °C for 10 s at the end. The qPCR results were visualized by Agilent Aria 1.8. Relative expression levels were normalized to those of GAPDH. Ct values obtained through RT-PCR can be found in Supplementary Table S3.

### Western blot analysis

Samples were lysed using NP-40 (50 mM Tris pH 8.0 containing 150 mM NaCl and 1% NP-40) and determined the protein concentration using BCA Protein Assay Kit (iNtRON Biotechnology, Inc.). Proteins then were separated on 12% sodium dodecyl sulfate–polyacrylamide gel electrophoresis (SDS-PAGE) gels and transferred to nitrocellulose membranes (BioRad Trans-Blot Turbo transfer system, Hercules, CA, USA). After blocked with blocking buffer, membranes were treated with primary antibodies (Supplementary Table S1) and incubated overnight at 4℃. HRP-conjugated goat anti-rabbit secondary antibody (Abcam) served as secondary antibodies were treated and incubated for 1 h at RT. ECL Reagent kit were treated before images of the western blot were taken using a ChemiDoc (ChemiDoc MB Imaging System, BioRad).

### Functional analysis of hiPSC-CMs using drug treatments

To visually analyze the drug responsiveness of the hiPSC-CM spheroids, changes in the phenomenon before and after drug administration were recorded as a video. Spheroids were treated with nifedipine and E4031 at concentrations of 50 nM and 100 nM for 15 min in incubator. After treatment, the cells were recorded for 1 min and the movements and blinking signals were analyzed using Fiji to observe drug-dependent changes.

### Non-spectroscopic video analysis of beating rate and contraction distance

For visually analyzing the beating period of hiPSC-cardiomyocytes, 10 µg/mL Ab-conjugated-RJPs were treated for 1 h before recorded for 1 min. Videos were captured by an inverted microscope (CKX3, Olympus, Japan) with camera (KCS3, Korea Lab Tech) at a resolution of 1,560 × 1,180 pixels and 10 fps using a 20 × objective and recorded using ToupView software (ToupTek, China). The movements and blinking signals of Ab-RJP were analyzed by Trackmate [41] plugin in Fiji to calculate the beating period and contraction magnitude of cardiomyocytes (Supplementary Fig. S2). At least 5 Ab-conjugated-RJPs at different positions around the spheroids were examined and analyzed before the final beating period of CMs were set.

### Scanning electron microscopic imaging

Silica micron-patterning array on the PAMCELL™ R100 plate and morphology of RJP were observed by field emission scanning electron microscope (JSM-7900F, JEOL Ltd., Seoul, South Korea).

Spheroids cultured on the PAMCELL™ R100 plate were prepared for SEM by cell silicification method [42]. After fixing with 4% PFA for 1 h and washing three times by DW, hiPSC-CM spheroids were incubated in a 100 mM Tetramethyl orthosilicate (TMOS, Sigma–Aldrich) solution in 1 mM Hydrochloric acid (HCl, Sigma–Aldrich) at 40℃ for at least 48 h. Spheroids then were dehydrated by sequential soaking in Ethanol 30%, 50%, 70%, 80%, 90%, and 100% in every 10 min. The surface of the hiPSC-CM spheroids was finally coated with Platinum, Pt before taking SEM.

### Statistical analyses

All statistical analyses were performed using GraphPad Prism 9 software (GraphPad, CA, USA). Data are shown as the mean ± standard error of the mean (SEM). Student’s *t*-test and one-way analysis of variance (ANOVA) were applied and a *p*-values or *p* ≤ 0.05, 0.01, 0.001, and 0.0001 were considered significant in all tests.

## Results

### PAMCELL™ R100 plate and RJPs fabrication

**Fig. 3:**
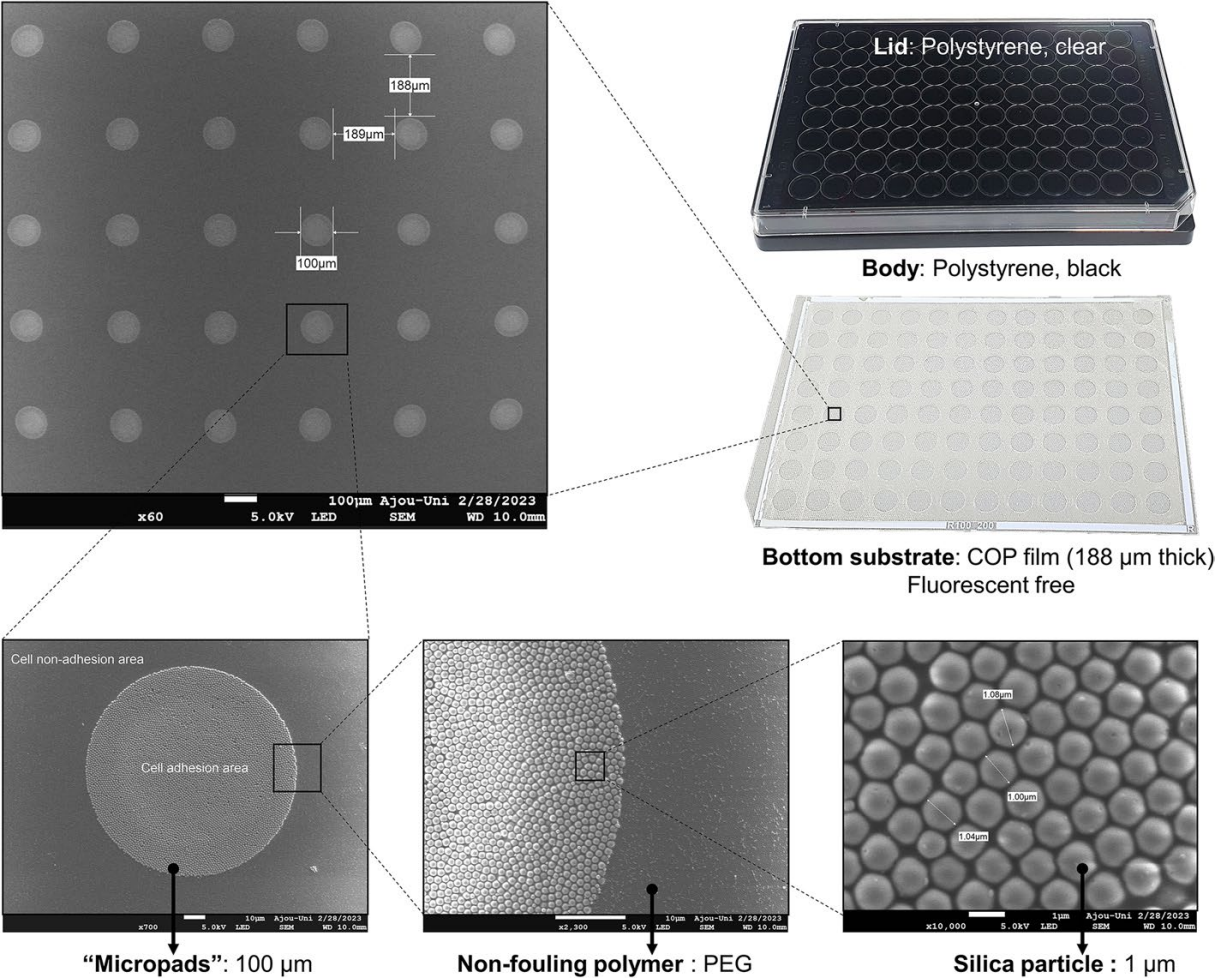
Components of the PAMCELL™ R100 plate and SEM images of “micro-pad” and silica particles

We have previously demonstrated that the PAMCELL™ R600 and R250 configuration suitable for cell migration, cell–cell cohesion, and spheroid formation of adipose-derived human mesenchymal stem cells [39]. However, the distinct behavior of hiPSC-CM, which are non-active and less prone to long-distance migration compared to mesenchymal stem cells, indicated that PAMCELL™ R100 would be more appropriate for inducing spheroid formation in these cells. In PAMCELL™ R100 plate, RGD-coated silica particles are densely arranged as a monolayer to form 100-µm diameter micropads, and each micropad is separated by approximately 200 µm (edge-to-edge; Fig. 3). Each well of 96-well plate contains about 350 micropads, where allows the cell adhesion, proliferation, aggregation to form size-defined spheroids. The area outside the micropads is covered with non-fouling polymer, PEG, to prevent cell adhesion. The combination of PEG (5 µm in height) and COP (188 µm in height) at the bottom of the substrate creates fluorescent-free areas (Fig. 1).

**Fig. 4:**
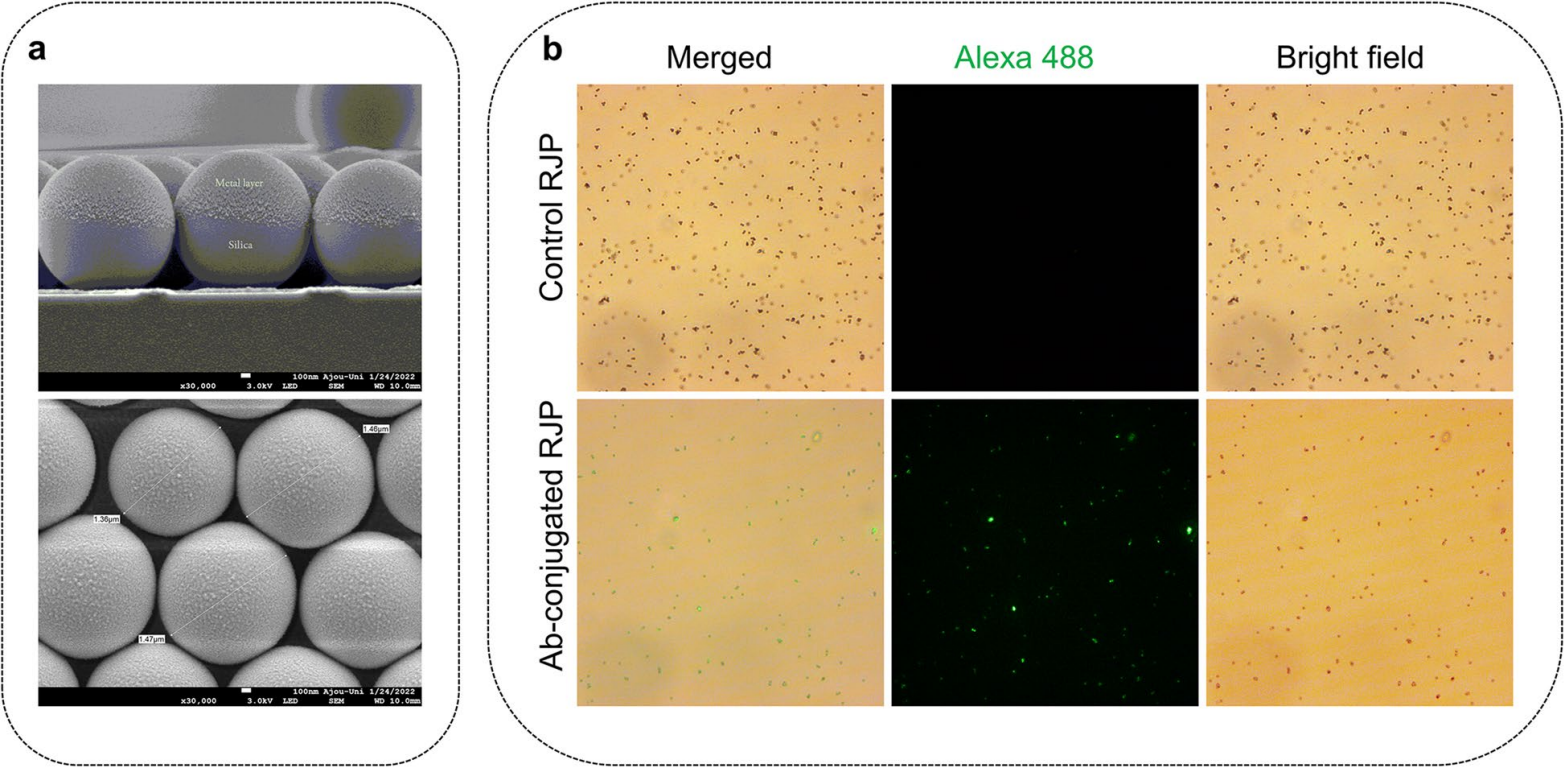
Characterization of RJPs. (**a**) SEM images of RJPs; (**b**) Fluorescence microscopy images of ab-conjugated RJP and control-RJPs after the labeling with Alexa fluor 488. Fluorescence signal was not observed in control-RJPs and large amounts of fluorescence signals were observed for ab-conjugated RJP

RJPs with diameters of 1.2 µm and 0.7 µm have been suggested as suitable optical probes and for cell surface modification previously [23, 24]. However, hiPSC-CM spheroid interacts better with larger RJPs, having a diameter of approximately 1.5 µm. SEM images confirmed the size and deposition of metal layers on the RJPs (Fig. 4a). To further enhance the interaction between RJPs and hiPSC-CMs spheroids, the metallic hemispherical part of the RJPs was selectively coated with anti-connexin 43 antibodies (CX43 Ab). Connexin 43, a gap-junction protein, plays a role in cell–cell communication, thus coating the RJPs with CX43 Ab was expected to strengthen the binding affinity of the RJPs and hiPSC-CMs spheroids. The Alexa Fluor 488-conjugated CX43 Ab coating on RJPs indicates successful Cx43 Ab coating on RJP surfaces, as the fluorescence signal coincided with RJPs (Fig. 4b).

### Formation of hiPSC-CM spheroids on PAMCELL™ R100 plate

**Fig. 5:**
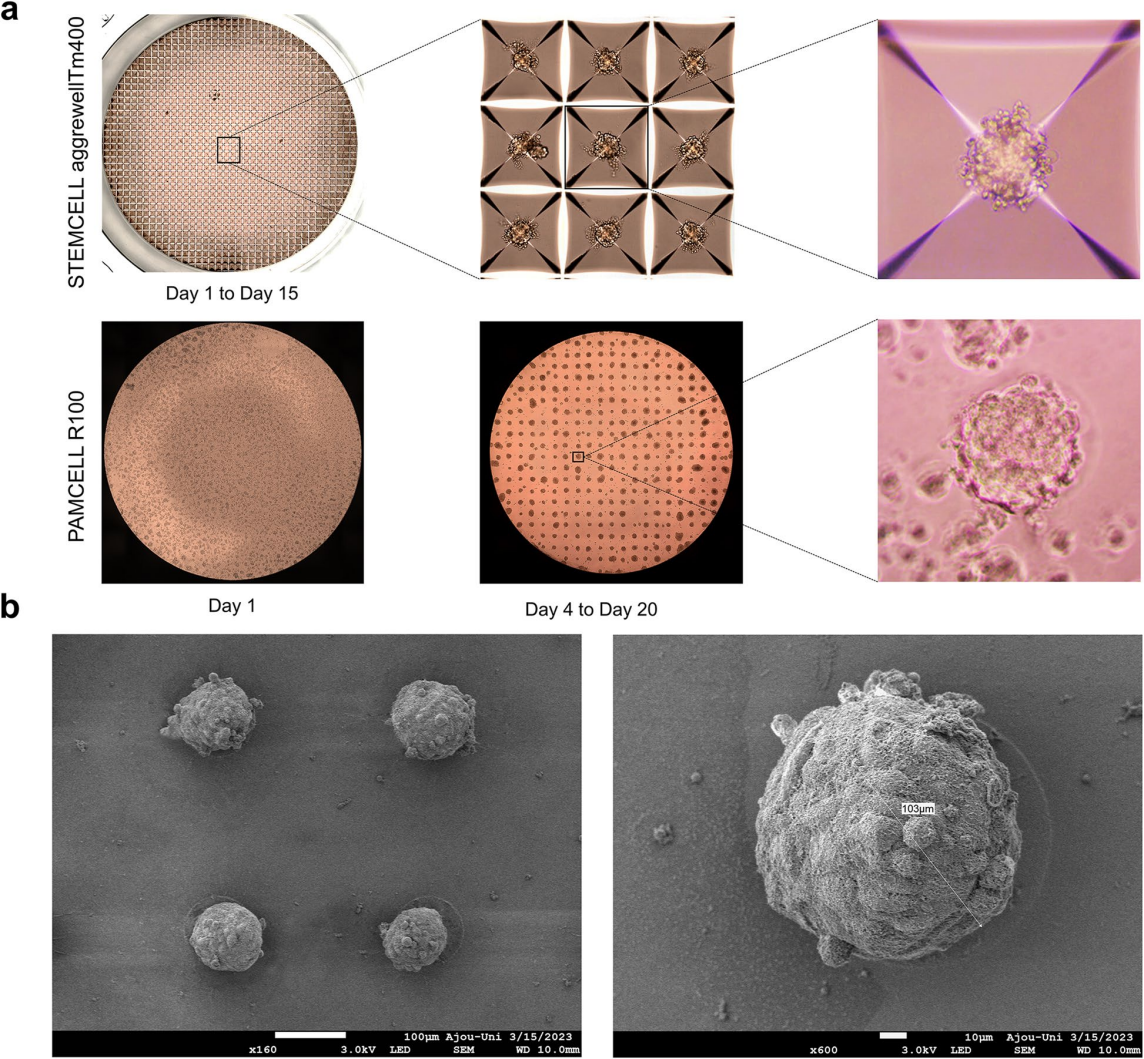
The formation of hiPSC-CM spheroids on the the STEMCELL AggreWell™ 400 and PAMCELL™ R100 plates. (**a**) Images of spheroids formed on two above mentioned plates; (**b**) SEM of spheroids grown on PAMCELL™ R100 plate

**Fig. 6:**
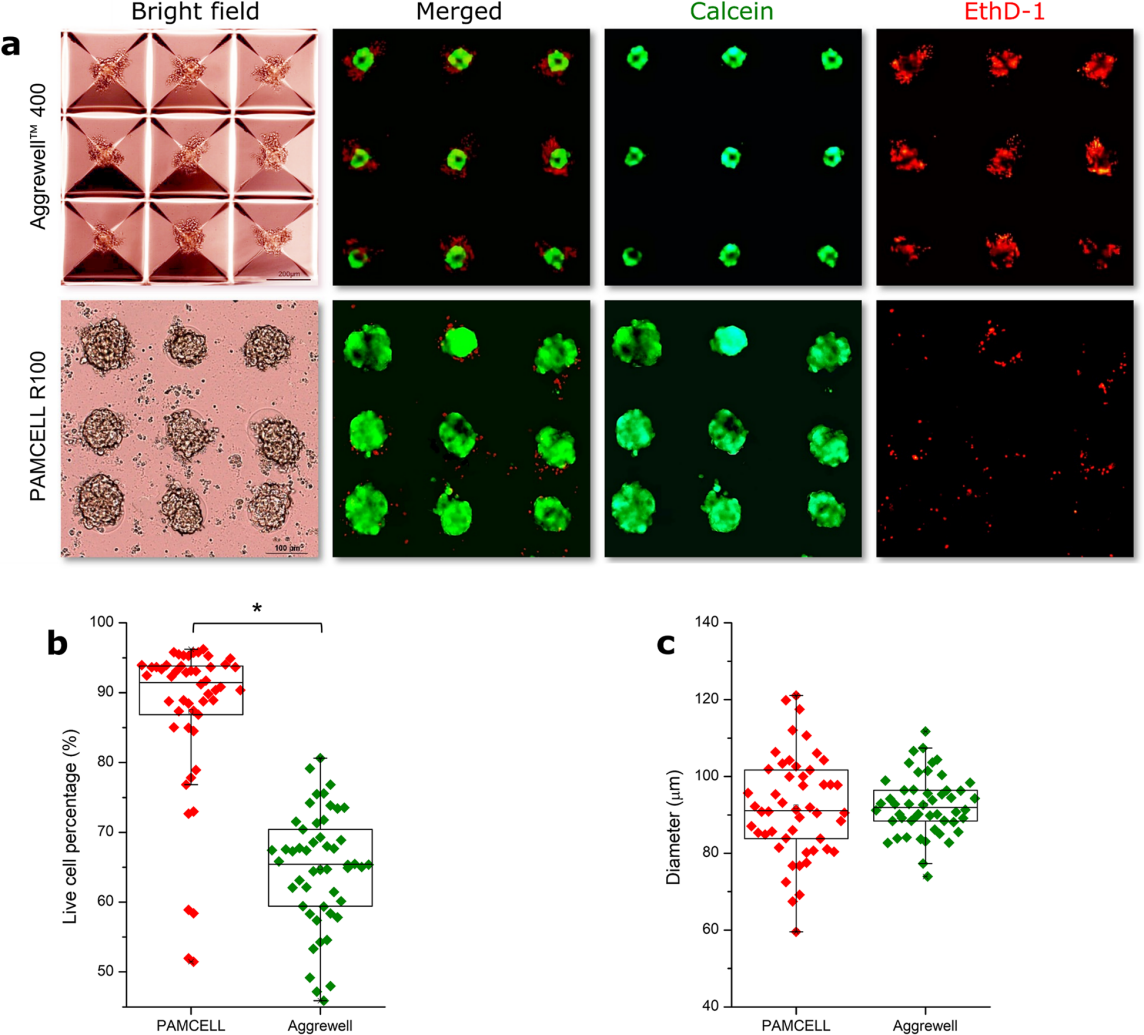
Live-dead assay to determine the viability of hiPSC-CM spheroids using C-AM and EthD-1. (**a**) Staining images of the spheroids grown on AggreWell™ 400 and PAMCELL™ R100 plate for C-AM (green), an indicator of live cells, and EthD-1 (red), an indicator of dead cells. Scale bar: 100 μm; (**b**) Verification of viability of hiPSC-CM spheroids through comparison of fluorescence (*n* = 50; * p ≤ 0.05); (**c**) Size of formed hiPSC-CM spheroids (*n* = 50) was measured by microscopy and analyzed by ImageJ

hiPSC-CMs resumed spontaneous beating within 2 days after thawing and attained a stable state after 5–7 days. A single synchronous monolayer of hiPSC-CMs formed readily on the pre-coated Matrigel layer. The iPSC-CM spheroid formation on the PAMCELL™ R100 plate was then monitored and compared with those in AggreWell™ 400. In the PAMCELL™ R100 culture platform, hiPSC-CMs settled and adhered to the areas with RGD-coated silica particles within 24 h. Nevertheless, it took up to 4 days for the cells to proliferate and form 3D-structured spheroids on the micropatterned pads. The average diameter of the spheroids was 92.27 µm, which was similar in size to those formed in the AggreWell™400 plates (Figs. 5 and 6**,** and Supplementary Table S4). These spheroids eventually lost their pulsatile function on day 20 of culturing (Fig. 5a). The morphology and structure of the hiPSC-CMs grown on the PAMCELL™ R100 plate was visualized using SEM imaging (Fig. 5b). Furthermore, a LIVE/DEAD assay was performed to assess the viability of hiPSC-CM spheroids (Fig. 6a). Interestingly, hiPSC-CM spheroids on PAMCELL™ R100 plate exhibited higher cell viability compared to those in AggreWell™ 400 at day 8 (87.39 ± 11.13% on the PAMCELL™ R100 plate and 64.89 ± 8.08% in the AggreWell™ 400; Fig. 6b).

These results suggest that spheroids formed in the reverse-pyramid-shaped space contained both live and dead cells, whereas spheroids formed on micropatterns of PAMCELL™ R100 predominantly consisted mostly of live cells.

### hiPSC cardiomyocyte bio-molecular functions

**Fig. 7:**
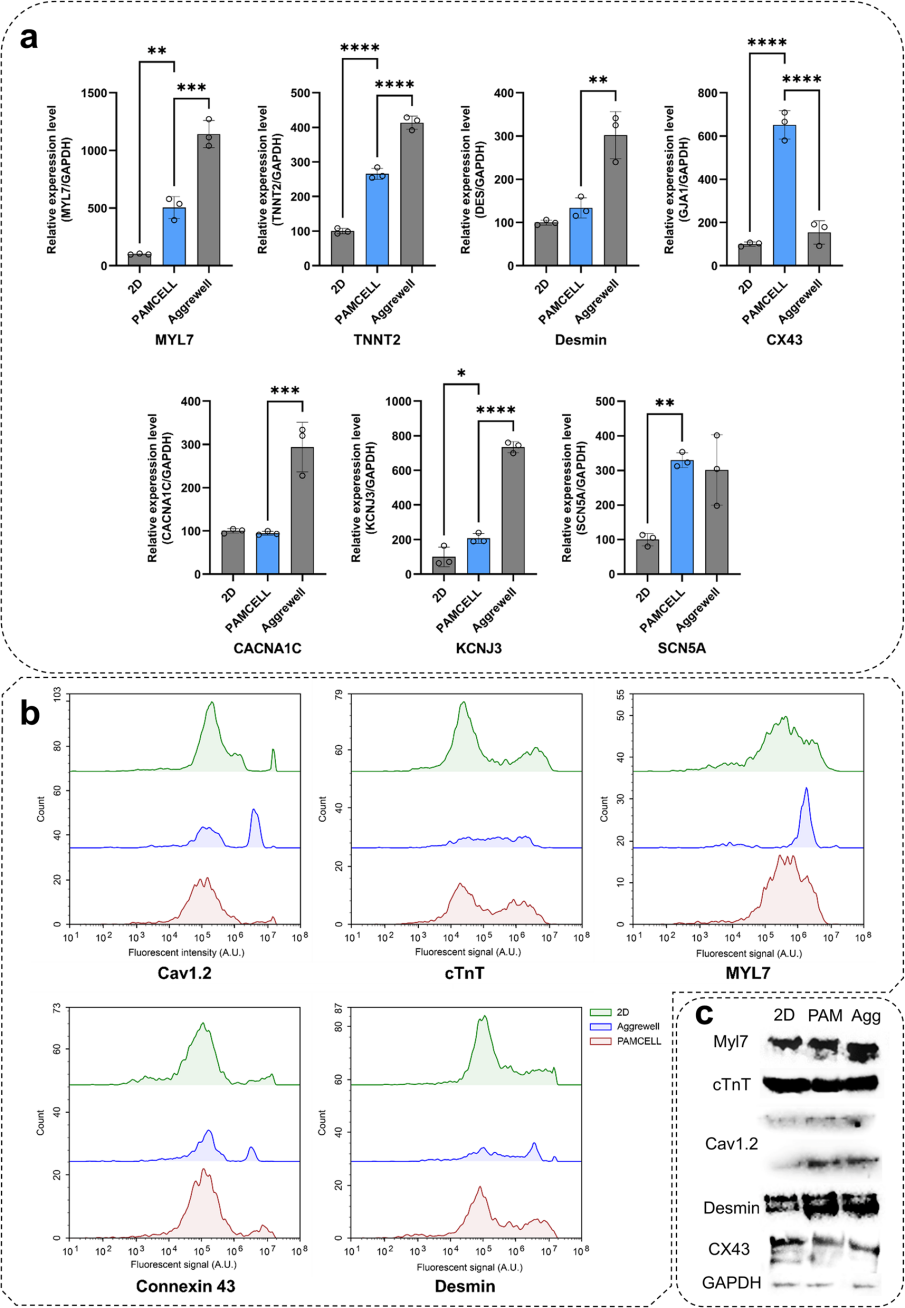
Quantification of of hiPSC 2D and 3D cardiomyocytes. (**a**) mRNA expression through real-time PCR analyses using primers for CM development markers (MYL7/TNNT2), gap junction/sarcomeric markers (CX43/DES), and the ion channel markers (CACNA1C/KCNJ3/SCN5A). Relative expression levels were normalized to those of GAPDH; (*n* = 3, *p ≤ 0.05, ** p ≤ 0.01, *** p ≤ 0.001, and ****p ≤ 0.0001); (**b**) 2D and 3D culture of hiPSC-CMs assessed by flow cytometric analysis; (**c**) Western blot experiments demonstrating the difference of the above markers in 2D vs. 3D cultures of hiPSC-CMs

We next evaluated the functionality of hiPSC-CMs generated on PAMCELL™ R100 plates (Fig. 7a). Initially, the mRNA expression levels of MYL7 and TNNT2 were significantly increased in hiPSC-CM spheroids compared to 2D monolayers, indicating a more mature phenotype in the spheroid structures. Additionally, increased Desmin expression in both PAMCELL™ R100 and AggreWell™ 400 spheroids suggested a more organized and mature CM phenotype. Notably, PAMCELL™ R100 spheroids exhibited 6.51 times higher CX43 expression compared to 2D hiPSC-CMs, suggesting efficient electrical signal propagation, while AggreWell™ 400 spheroids showed a 1.54-fold increase compared to 2D hiPSC-CMs. Furthermore, we examined the electrophysiological characteristics of 2D CMs and 3D spheroids (Fig. 7a). Although the mRNA expressions of the calcium ion channel (CACNA1C) and potassium ion channel (KCNJ3) in PAMCELL™ R100 spheroids were comparable to those in 2D CMs, SCN5A expression in PAMCELL™ R100 spheroids was significantly upregulated, reaching similar levels to those observed in AggreWell™ 400 spheroids. This finding suggests that SCN5A may play a crucial role in electric signal propagation in PAMCELL™ R100 spheroids.

**Fig. 8:**
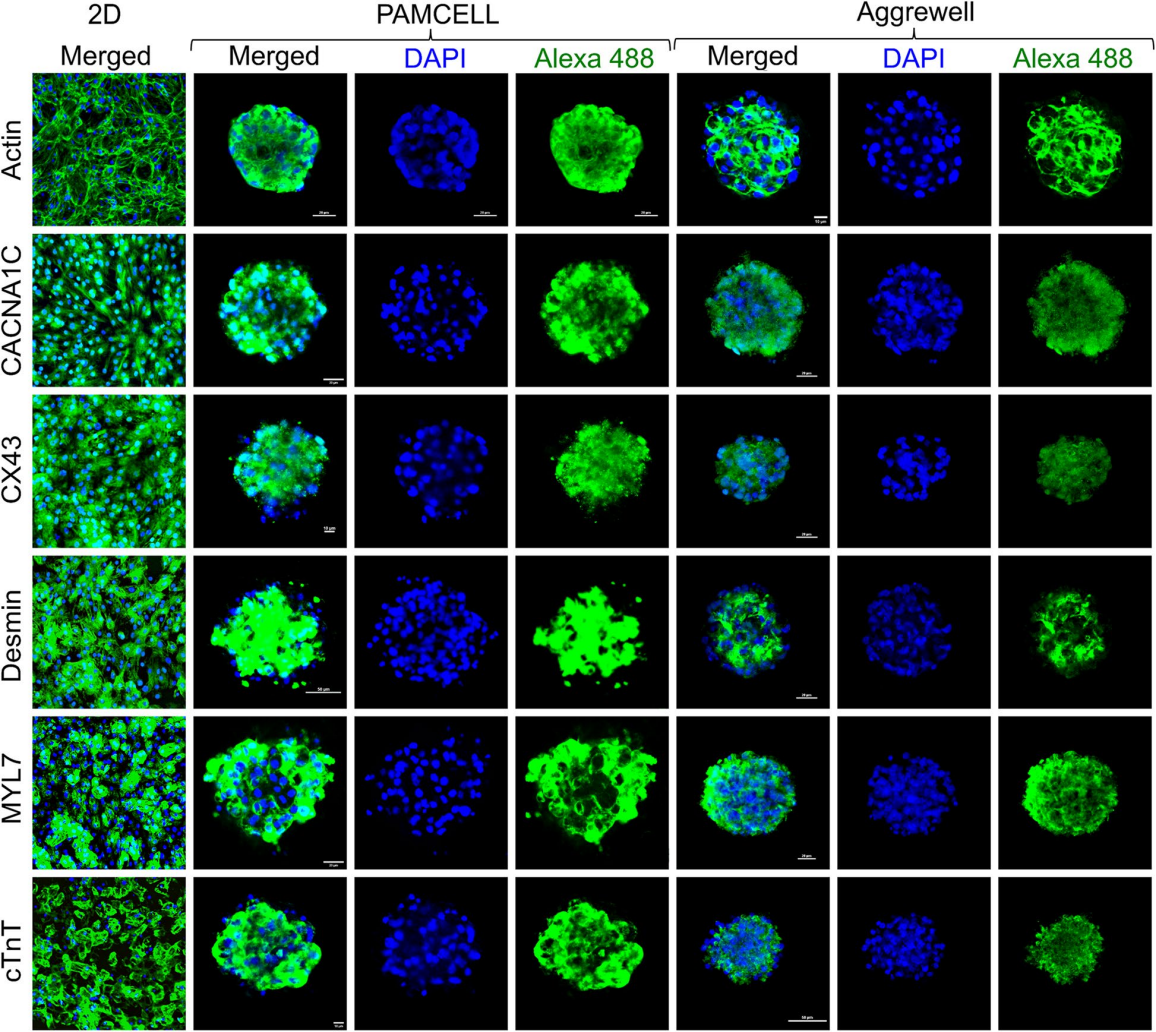
Immunostaining of hiPSC 2D and 3D CMs CMs with F-actin, anti- CACNA1C, anti-CX43, anti-Desmin, anti-MYL7, and anti-cTnT. Nuclei are labeled with DAPI (blue) and scale bars are included in images

Subsequently, protein expression was assessed by western blot, FACS analyses and immunofluorescence staining after 8 days of hiPSC-CM culture (Fig. 7b, c). High expression levels of Cav1.2, cTnT, Myl7, Cx43, and Desmin were observed across all platforms, with over 85% of hiPSC-CMs expressing these markers and no significant differences between groups. This result corroborates the mRNA expression, indicating stable and differentiated CMs in 3D platforms. Immunofluorescence staining, however, revealed significantly higher cardiac, skeletal, and ion channel marker expression in 3D cultures compared to 2D hiPSC-CM monolayers (Fig. 8).

In summary, the functionality of hiPSC-CM spheroids generated on PAMCELL™ R100 revealed more mature phenotypes, efficient electrical signal propagation, and increased expression of crucial ion channels in 3D spheroids compared to 2D monolayers.

### Beating periodicity and rhythm recorded from MCs and RJPs interaction

To assess cardiac rhythms of hiPSC-CMs spheroids, we first examined the intracellular Ca^2+^ transients in PAMCELL™ R100 spheroids using fluorescent Ca^2+^ dye and confocal microscopy, recording spontaneous beating (Supplementary Video S1). Synchronized, robust Ca2 + transients were observed during stable beating were observed.

**Fig. 9:**
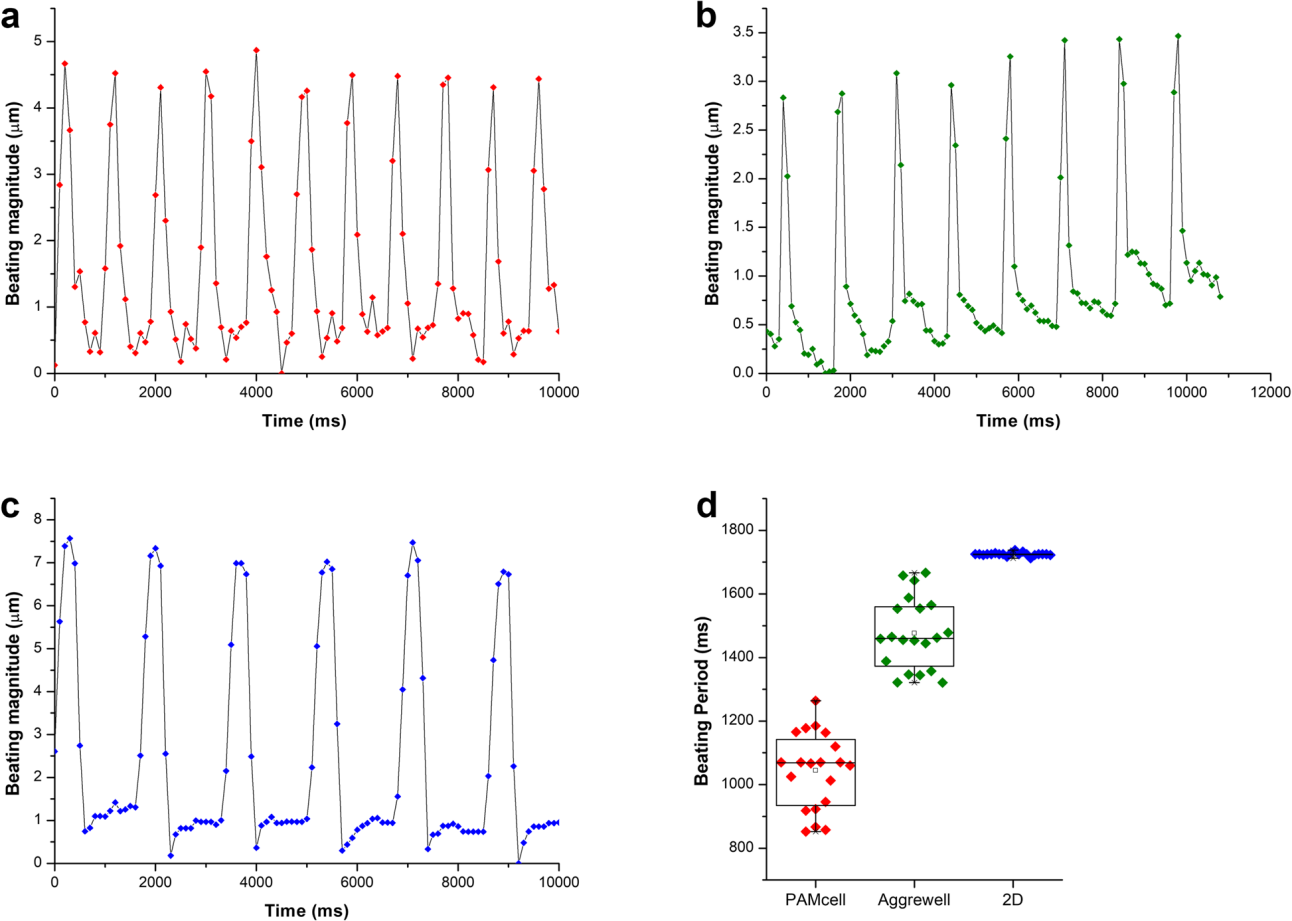
The difference of the beating rhythm of hiPSC-CMs between 2 and 3D culture. (**a**) Beating rhythm of hiPSC-CMs in PAMCELL™ R100; (**b**) Beating rhythm of hiPSC-CMs in AggreWell™ 400; (**c**) Beating rhythm of hiPSC-CMs in 2D platform; (**d**) The beating period of hiPSC-CMs in 2D and 3D cells analyzed (*n* = 20)

Subsequently, RJP was employed to evaluate cardiac rhythms of hiPSC-CMs following an 8-day culture period, examining 20 spheroids and cells across various platforms by monitoring the movements and blinking signals of RJPs interacting with cell and spheroid surfaces (Supplementary Fig. S3, Supplementary Video S2–5). Ab-conjugated RJPs were evenly distributed on the top of the surface of the spheroids and 2D cells (Supplementary Fig. S4 and Supplementary Video. S6, 7), facilitating signal collection and analysis that reflected the beating patterns of the spheroids and cells (Supplementary Table S5). The findings showed consistence with results from other CM monitoring methods, including electrophysiological recording, patch-clamp measurement [17, 43, 44], and Ca^2+^ current recording [17, 43, 45, 46] (Fig. 9).

In summary, RJP effectively evaluated cardiac rhythms in hiPSC-CMs, demonstrating compatibility with established recording techniques.

### hiPSC cardiomyocyte spheroid responds to drugs analyzed by RJPs

**Fig. 10:**
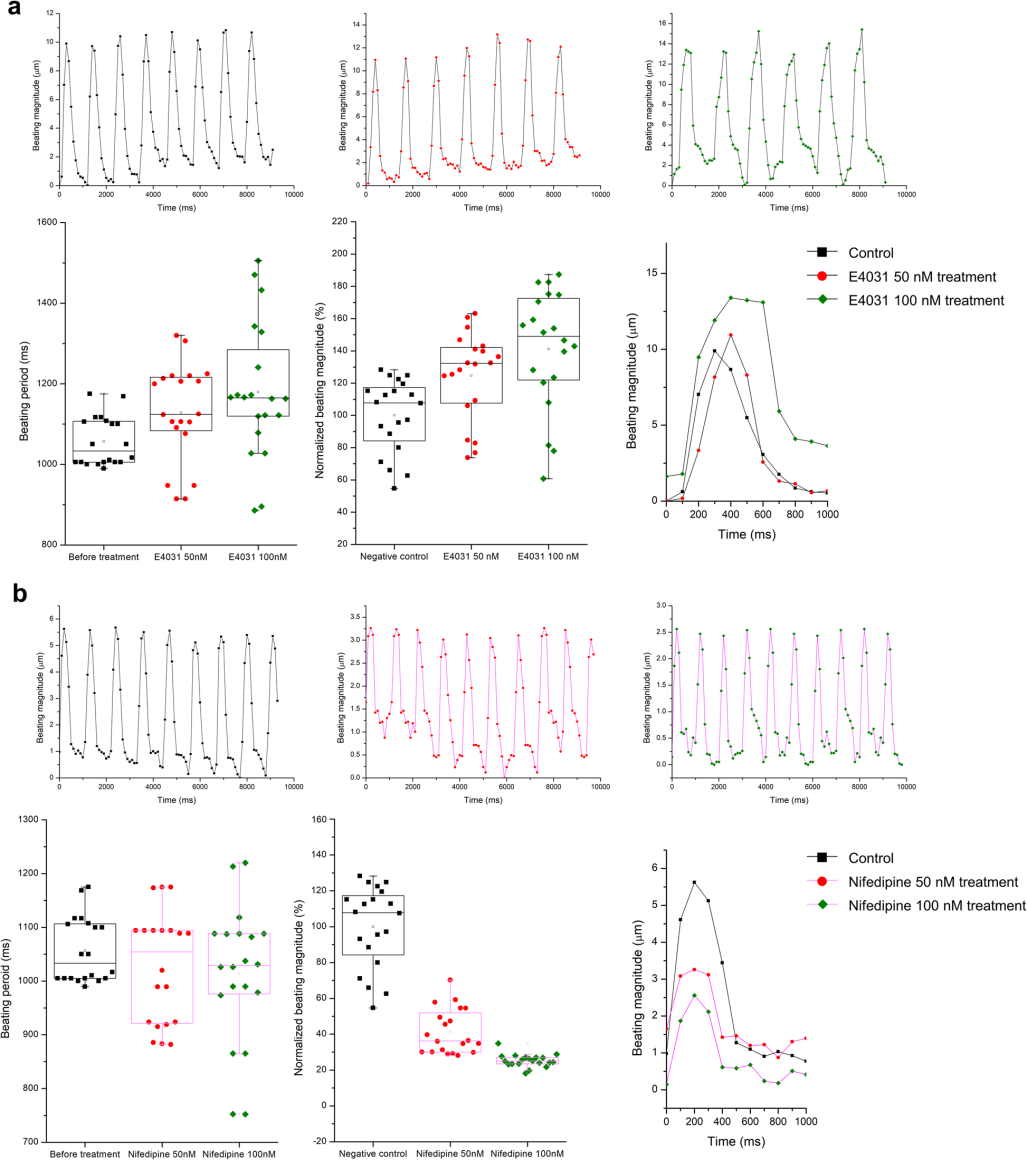
The beating rhythm of hiPSC-CMs under effect of inotropic drugs. (**a**) Beating rhythm of hiPSC-CMs under effect of E4031. (**b**) Beating rhythm of hiPSC-CMs under effect of Nifedipine

Finally, we evaluated the effectiveness of RJP-based CM beating monitoring system in reflecting the changes in cardiac beating patterns induced by positive and negative inotropic drugs. The PAMCELL™ R100 platform was utilized to produce uniform-sized hiPSC-CM spheroids that function over longer period, as shown in Fig. 5. The changes in beating behavior were recorded by monitoring the deflection of RJPs on PAMCELL™ R100 spheroids after exposure to the drugs. As anticipated, E4031, a K^+^ channel blocker, lengthened the beating frequency and amplified the magnitude (Fig. 10a), while Nifedipine, a Ca2 + channel blocker, reduced both beating frequency and magnitude (Fig. 10b). These results are consistent with previous studies [17, 47, 48] and further validated the functionality of our image-based analysis method in evaluating changes in the beating behavior of CMs under external stimuli, supporting its validity for measuring cardiac rhythms.

## Discussion

The 3D culture models of cardiac stem cells has been developed for decades to overcome the intrinsic limitations 2D cell cultures [14]. Although the 3D micropattern platform in this study is not novel and previously reported from other groups [39, 49, 50], it is the first attempt to test hiPSC-CM spheroid culture on semi-adhesive surface. The choice of PAMCELL™ R100 for this study was guided by the specific needs of hiPSC-CMs. Unlike cells like cancer cell lines that display extensive migratory capacity, hiPSC-CMs exhibit less activity and long-distance migration. Thus, the R100 motif of the PAMCELL™ platform, coupled with a well-designed microplate configuration, ensures each spheroid maintains optimal conditions for growth and function. The configuration was meticulously designed to accommodate approximately 100,000 cells, forming 350 spheroids each containing around 285 cells, an optimal cell density for hiPSC-CMs.

Results confirm the PAMCELL™ R100 plate’s superiority in long-term culture, where significant differences in spheroid viability were observed between the confined reverse-pyramid-shaped space (AggreWell™ 400) and micro-patterned technology (PAMCELL™ R100; Fig. 5). Spheroids in AggreWell™ 400 contained both dead and live cells, likely due to limited oxygen and nutrient supply (Fig. 6). In contrast, PAMCELL™ R100 spheroids mainly contained live cells, possibly due to the controlled environment provided by micropatterns. Thus, PAMCELL™ R100 platform may be more suitable for generating spheroids with higher viability for drug discovery and tissue engineering applications than AggreWell™ 400 platform.

PAMCELL™ R100 platform, allowing for the simultaneous culture and monitoring of multiple spheroids within the same well, enhances the statistical roboustness of our findings and reduces the risk of false positive or negatives. External factors such as pH and temperature can significantly influence the behavior of hiPSC-CMs. As demonstrated previously, the beating frequency of iPSC-CMs can be halved at 32℃ compared to physiological conditions [51]. Additionally, hosting multiple aggregates in the same well, which are exposed to an identical medium, assists in maintain the uniformity of the treatment. This approach curtails the variability potentially arising from individual treatments for each aggregate. Notably, this experimental configuration might limit the discernment of effect produced by soluble factors among aggregates, which merits consideration in future studies.

Indeed, PAMCELL™ R100 spheroids displayed significant variations in the beating frequency and magnitude upon treatment with ion channel blockers (Fig. 10). These variations can be attributed to two main factors: chemical and mechanical signals given to cells. First, the expression of ion channels, which play a crucial role in regulating the electrical activity of cardiac cells, may vary based on culture conditions (Fig. 7a). Previous research has demonstrated that changes in ion channel expression can affect the beating behavior of cardiac cells [43, 52]. Second, different culture substrates can lead to variations in the mechanical properties of cells, such as the stiffness of the extracellular matrix and the rate of cell proliferation, which may also influence beating behavior [53, 54]. Additionally, the presence of dead cells in the AggreWell™400 system and the propagation of beating signals over large surface areas can contribute to differences in beating patterns across various platforms.

Building on the observed variations in beating patterns, the use of retroreflective light, known for its unique property of reflecting light back to the source direction, offers a promising approach to monitor CM behavior. Recently, retroreflective Janus particles (RJPs) have emerged as a promising optical probe for monitoring macrophage migration [24] and *Salmonella Typhimurium* tracking [25]. Here, RJPs are functionalized with anti-CX43 Abs, enabling them to specifically bind to gap junction proteins on cardiomyocyte surfaces. Upon introduction to a cardiomyocyte culture, RJPs bind to Cx43 proteins on cell membranes, synchronizing their motion with the cardiomyocytes' beating rhythm. As CMs contract and relax, RJPs respond by moving and reflecting light back to the detector, generating a retroreflective signal for real-time monitoring of cellular beating behavior. This specific binding allows RJPs to move in tandem with cardiomyocyte beating, producing a detectable retroreflective signal using a microscope equipped with a retroreflective detection system.

In contrast to conventional methods for measuring CM contraction, which involve using fluorescent Ca^2+^ indicators like Fura-2 or Indo-1, RJPs present a more viable alternative. The use of fluorescent Ca^2+^ indicators has several drawbacks: they require expensive equipment and near-violet excitation wavelengths that can cause long-term damage to cells, and the charged nature of Fura-2 can limit its ability to cross cell membranes, necessitating the use of nonionic surfactants to aid in cellular penetration [17]. These limitations make fluorescent Ca^2+^ indicators less than ideal for sensitive cell lines such as hiPSC-CMs. As demonstrated in previous studies, RJPs do not harm cells and can be safely used for long-term experiments [24]. Additionally, the particles are cost-effective and easy to produce, making them a practical choice for large-scale studies. RJPs offer high sensitivity and specificity, as they are designed to respond to changes in the local refractive index, allowing for precise measurements of cardiomyocyte beating behavior. The retroreflective signal generated by these particles is highly stable, enabling accurate and reliable detection of changes in beating behavior over extended periods of time. However, RJPs do have some limitations: their size may limit penetration into certain types of cells or tissues, and their retroreflective signal is angle-dependent, potentially affecting measurement accuracy. Furthermore, while RJPs are non-toxic, there may be concerns about their long-term biocompatibility and potential immunogenicity in vivo. Despite these limitations, this study demonstrates the promising use of RJPs as an optical probe for detecting the beating rhythm of cardiomyocytes and developing new therapies for cardiovascular disease.

## Conclusion

Here, we successfully demonstrated hiPSC-CM spheroid formation using PAMCELL™ R100 for a period of 20 days. A 96-well PAMCELL™ R100 plate was designed with micropatterned silica beads to achieve uniform spheroids with an average diameter of 90 µm. The PAMCELL™ R100 spheroids retained the characteristics and functions of cardiac stem cells, with higher expression levels of cardiac-specific gene and protein expressions compared to 2D cultured hiPSC-CMs. Our study also introduced a simple and effective monitoring system for measuring heart rhythm, based on the reflective properties of RJP particles, using only a camera for reverse imaging and a white light source. This system accurately detected changes in the beating rhythm of cardiac cells upon treatment with drugs that target intracellular ion channels.

In summary, this study provides a novel approach to the spheroid formation and drug testing of cardiac stem cells, which is crucial for the development of laboratory-level diagnostic and therapeutic methods for new cardiac diseases. The results of this study advance stem cell research and offer new insights into the use of 3D culture platforms for developing cardiovascular disease treatments.

### Supplementary Information

**Additional file 1: Fig. S1. **Peptide conjugated silica particle characterization on PAMCELL^TM^ R100 plate. **Fig. S2.** Schematic diagram of non-spectroscopic video analysis of beating contraction. **Fig. S3.** RJP distribution on the surface of cardiomyocytes. ** Fig. S4.** RJP movement in 20 frame with each frame taken at an interval of 24 fps. **Table S1.** Antibodies used in the present study. **Table S2.** Primers used in the present study. **Table S3.** Raw data for the real-time PCR analyses. **Table S4.** Size distribution of 3D iPSC-cardiomyocyte spheroids grown on Aggrewell and PAMCELL^TM^ plate. **Table S5.** Beating period of iPSC-cardiomyocytes on 2D platform and, Aggrewell^TM^ and PAMCELL^TM^ plate.

**Additional file 2: Video S1.** The presence of Ca2 transients of a spheroids grown on the PAMCELL^TM^ plate.

**Additional file 3: Video S2.** Retroreflective signal of hiPSC-CMs in PAMCELL^TM^ before analysis.

**Additional file 4: Video S3.** Retroreflective signal of hiPSC-CMs in PAMCELL^TM^ after analysis.

**Additional file 5: Video S4.** Retroreflective signal of hiPSC-CMs in Aggrewell before analysis.

**Additional file 6: Video S5.** Retroreflective signal of hiPSC-CMs in Aggrewell after analysis.

**Additional file 7: Video S6.** Retroreflective signal of hiPSC-CMs in 2D culture before analysis.

**Additional file 8: Video S7.** Retroreflective signal of hiPSC-CMs in 2D culture after analysis.

## Data Availability

The datasets used and/or analyzed during the current study are available from the corresponding author upon reasonable request.

## Abbreviations

2D: Two-dimensional monolayer
3D: Three-dimensional
Ab-Conjugated RJPs: Antibody conjugated RJPs
Anti-CX43 ab: Anti-connexin 43 antibody
BSA: Bovine serum albumin
CAMM: Cardiosight-S Advanced Maintenance Medium
CAPM: Cardiosight-S Advanced Plating Medium
C-AM: Calcein AM
CDI: Carbonyldiimidazole
COP: Cyclo Olefin Polymer
CM: Cardiomyocytes
CVD: Cardiovascular diseases
DAPI: 4′,6-Diamidino-2-phenylindole dihydrochloride
EDC/NHS: N-ethyl-N′-(3-(dimethylaminopropyl)carbodiimide/N-hydroxysuccinimide
EthD-1: Ethidium homodimer-1
ECM: Extracellular matrix
FACS: Flow cytometry
FBS: Fetal bovine serum
HCl: Hydrochloric acid
hiPSC-CMs: Human induced stem cell-cardiomyocytes
PBST: Phosphate-Buffered Saline in 0.05% Tween
PET: Polyethylene terephthalate
PLGA: Poly (lactic-co-glycolic acid)
RGD: Arginyl-glycyl-aspartic acid
RJP: Retroreflective Janus Microparticles
SDS-PAGE: Sodium dodecyl sulfate–polyacrylamide gel electrophoresis
SEM: Scanning Electron Microscopy
TMOS: Tetramethyl orthosilicate
