# Supplementary material for: A novel and cost-effective method for high-throughput 3D culturing and rhythmic assessment of hiPSC-derived cardiomyocytes using retroreflective Janus microparticles
Source: Biomater Res. 2023 Aug 16;27:79. doi: 10.1186/s40824-023-00416-4 (PMC10428620; doi:10.1186/s40824-023-00416-4)
Supplement: Supplementary file 1 — Additional file 1: Fig. S1. Peptide conjugated silica particle characterization on PAMCELLTM R100 plate. Fig. S2. Schematic diagram of non-spectroscopic video analysis of beating contraction. Fig. S3. RJP distribution on the surface of cardiomyocytes. Fig. S4. RJP movement in 20 frame with each frame taken at an interval of 24 fps. Table S1. Antibodies used in the present study. Table S2. Primers used in the present study. Table S3. Raw data for the real-time PCR analyses. Table S4. Size distribution of 3D iPSC-cardiomyocyte spheroids grown on Aggrewell and PAMCELLTM plate. Table S5. Beating period of iPSC-cardiomyocytes on 2D platform and, AggrewellTM and PAMCELLTM plate. [file 40824_2023_416_MOESM1_ESM.docx]

**Supplementary Materials**

**A novel and cost-effective method for high-throughput 3D culturing and rhythmic assessment of hiPSC-derived cardiomyocytes using** **retroreflective Janus microparticles**

Huyen T. M. Pham^1†^, Duc Long Nguyen^1†^, Hyo-Sop Kim^1^, Eun Kyeong Yang^1^, Jae-Ho Kim^1*^, Hyun C. Yoon^1*^, Hyun-Ji Park^1*^

^1^Department of Molecular Science and Technology, Ajou University, Suwon, 16499, South Korea

^†^These authors have contributed equally to this work.

^*^Corresponding authors’ e-mail:

hyunjipark@ajou.ac.kr, hcyoon@ajou.ac.kr, jhkim@ajou.ac.kr


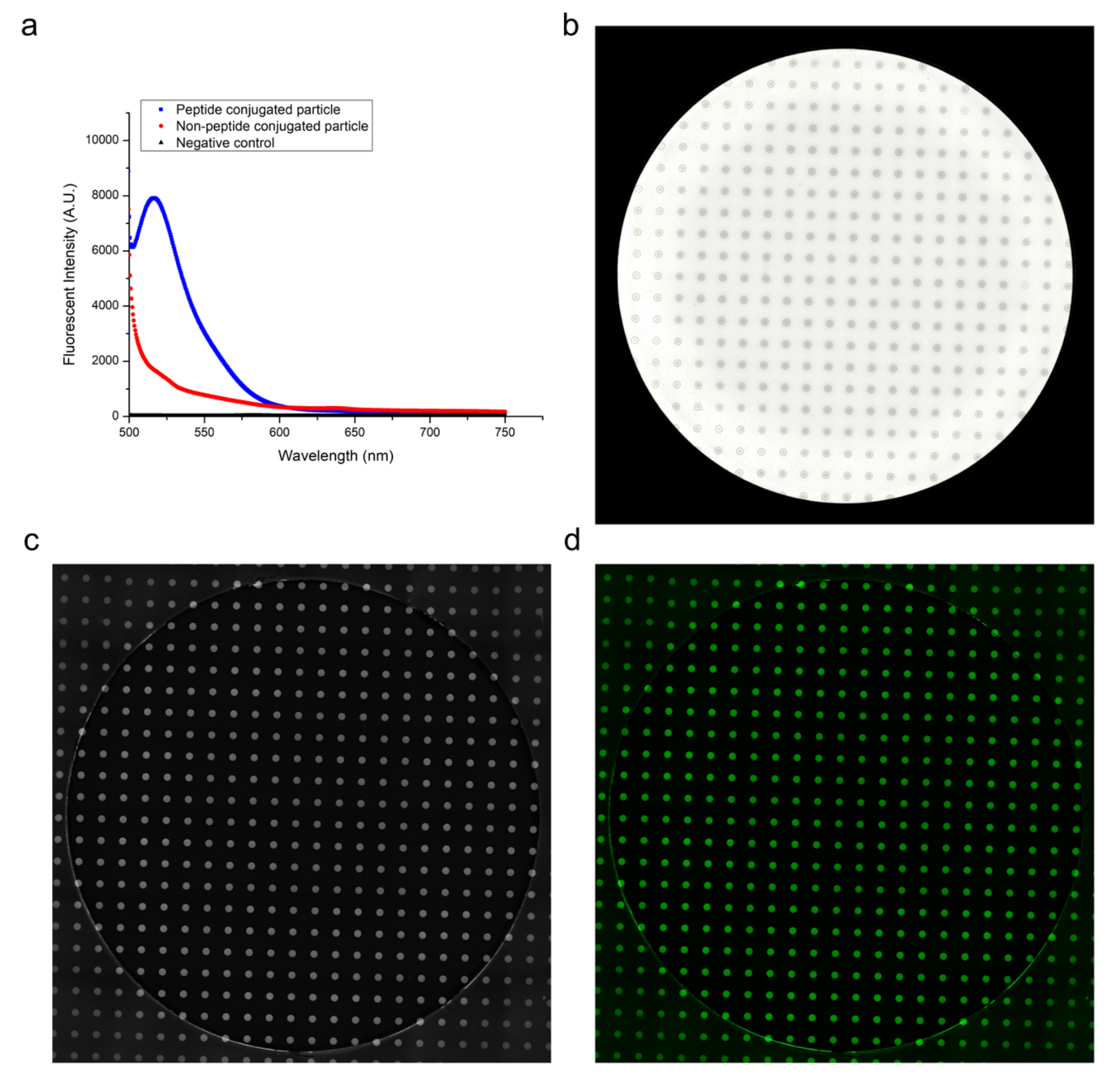


**Supplementary Figure S1.** Peptide conjugated silica particle characterization on PAMCELLTM R100 plate. **(a)** Fluorescence intensity plots of peptide conjugated silica particle (FITC-RGD conjugated Silica particles in HEPES buffer solution), non-peptide conjugated particle (amine modified Silica particles in HEPES buffer solution), and negative control (HEPES buffer solution); **(b)** Bright field image of a PAMCELLTM R100 plate well; **(c)** Fluorescence microscopy images of non FITC-RGD conjugated silica particles on a PAMCELL plate well; **(d)** Fluorescence microscopy images of FITC-RGD conjugated silica particles on a PAMCELLTM R100 plate well.


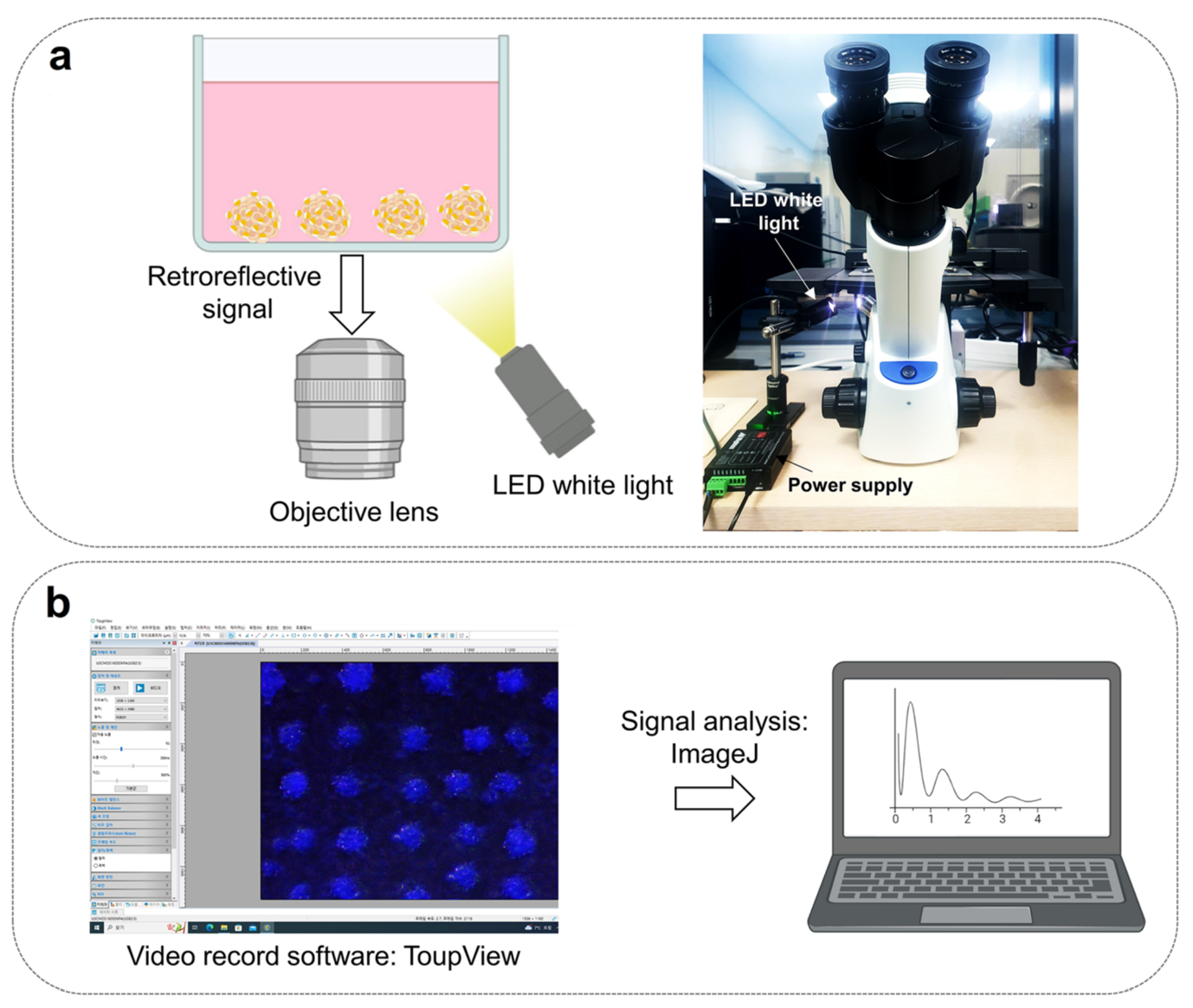


**Supplementary Figure S2.** Schematic diagram of non-spectroscopic video analysis of

beating contraction. **(a)** Nonspectroscopic video of cardiomyocyte contraction recording via

blinking signal of RJP; **(b)** Signal analysis.


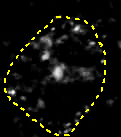

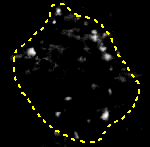

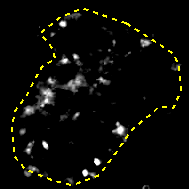


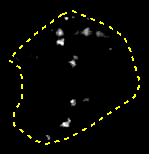

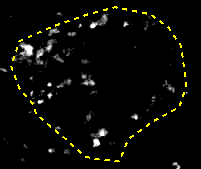

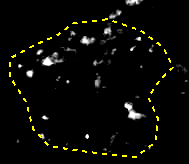


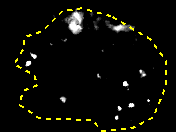

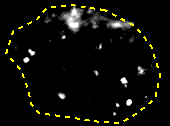


**Supplementary Figure S3. RJP distribution on the surface of cardiomyocytes.**


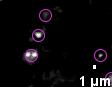

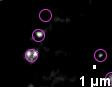

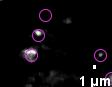

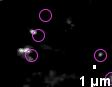

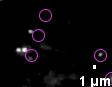


| 1 | 2 | 3 | 4 | 5 |
| --- | --- | --- | --- | --- |
|  |  |  |  |  |


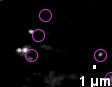

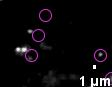

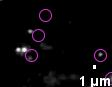

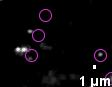

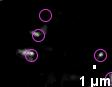


| 6 | 7 | 8 | 9 | 10 |
| --- | --- | --- | --- | --- |


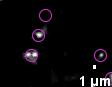

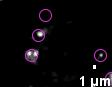

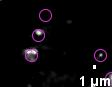

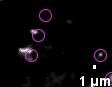

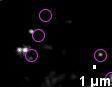


| 11 | 12 | 13 | 14 | 15 |
| --- | --- | --- | --- | --- |


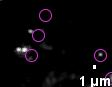

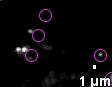

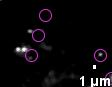

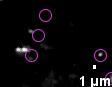

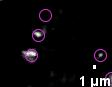


| 16 | 17 | 18 | 19 | 20 |
| --- | --- | --- | --- | --- |

**Supplementary Figure S4. RJP movement in 20 frame with each frame taken at an**

**interval of 24 fps.**

**Supplementary Table S1. Antibodies used in the present study.**

| **Name** | **Type of Ab** | **Company** | **Catalog** | **Host animal** | **Dilution rate for WB** | **Dilution rate for FACS** | **Dilution rate for ICC** |
| --- | --- | --- | --- | --- | --- | --- | --- |
| Anti-cTnT | 1^st^ | Abcam | ab209813 | Rabbit | 1/5000 | 1/500 | 1/1000 |
| Anti-MYL7 | 1^st^ | Abcam | ab127001 | Rabbit | 1/1000 | 1/200 | 1/200 |
| Anti-CNCNA1C | 1^st^ | Thermo Fisher | PA5-77297 | Rabbit | 1/200 | 1/100 | 1/500 |
| Anti-Desmin | 1^st^ | Cellsignal | D93F5 | Rabbit | 1/1000 | 1/200 | 1/200 |
| Anti-Connexin 43 | 1^st^ | Cellsignal | E7N2R | Rabbit | 1/1000 | 1/400 | 1/400 |
| Goat Anti-Rabbit IgG H&L | 2^nd^ | Abcam | ab6721 | Goat | 1/2000 | ̶ | ̶ |
| Alexa Fluor™ 488, Goat Anti-Rabbit igG | 2^nd^ | Thermo Fisher | A-11008 | Goat | ̶ | 1/500 | 1/200 |

**Supplementary Table S2.** **Primers used in the present study.**

| **Gene** | **Encoded protein** |  | **Sequences** | **Tm (℃)** |
| --- | --- | --- | --- | --- |
| *SCN5A* | SCN5A | FW | 5'-CAT CTG CGT CAT GCT CAT TGG C-3' | 68 |
|  |  | RV | 5'-TCT GGT GGA AGC GGA TGA ACT C-3' | 68 |
| *KCNJ3* | KCNJ3 | FW | 5'-GAT CTC CAT GAG GGA CGG AAA AC-3' | 67 |
|  |  | RV | 5'-GAA GGA ACT CAC CCT CAG GTG T-3' | 69 |
| *CACNA1C* | CACNA1C | FW | 5'-CGT TCT CAT CCT GCT CAA CAC C-3' | 68 |
|  |  | RV | 5'-GAG CTT CAG GAT CAT CTC CAC TG-3' | 67 |
| *TNNT2* | cTnT | FW | 5'-AAG AGG CAG ACT GAG CGG GAA A-3' | 71 |
|  |  | RV | 5'-AGA TGC TCT GCC ACA GCT CCT T-3' | 71 |
| *MYL7* | MYL7 | FW | 5'-CGA CCA GAA TCG TGA TGG CAT C-3' | 68 |
|  |  | RV | 5'-CAA AGA GCG TGA GGA AGA CGG T-3' | 69 |
| *DES* | Desmin | FW | 5'-TCC AGT CCT ACA CCT GCG AGA T-3' | 70 |
|  |  | RV | 5'-CGC AAT GTT GTC CTG GTA GCC A-3' | 70 |
| *GJA1* | Connexin43 | FW | 5'-GGA GAT GAG CAG TCT GCC TTT C-3' | 68 |
|  |  | RV | 5'-TGA GCC AGG TAC AAG AGT GTG G-3' | 69 |

**Supplementary Table S3. Raw data for the real-time PCR analyses.** The Ct for each mRNA and endogenous control GAPDH in each sample were used to create ΔCt values [Ct(mRNA) – Ct(GAPDH)]. The relative quantification (R) was calculated using the equation: R= 2−ΔΔCt. AVG means average.

| Gene |  | **MYL7** | **cTnT** | **Desmin** | **Conexin 43** | **CACNA1C** | **KCNJ3** | **SCN5A** |
| --- | --- | --- | --- | --- | --- | --- | --- | --- |
|  | 2D | 100 | 100 | 100 | 100 | 100 | 100 | 100 |
| Relative expression level | PAMCELL | 504.377662 | 266.1311999 | 133.9955818 | 651.5657302 | 94.58857897 | 207.2683 | 330.0222 |
|  | Aggrewell | 1142.42397 | 413.5838688 | 302.160469 | 154.1730025 | 293.9321386 | 734.6818 | 301.6355 |

|  | **GAPDH (Ct)** | **AVG(GAPDH)** | **MYL7 (Ct)** | **ΔCt MYL7** | **R** | **AVG (R)** |
| --- | --- | --- | --- | --- | --- | --- |
| **2D** | 18.14 | 18.15 | 21.2 | 3.05 | 0.120742 | 0.124513 |
|  | 18.17 |  | 21.08 | 2.93 | 0.131215 |  |
|  | 18.14 |  | 21.19 | 3.04 | 0.121582 |  |
| **PAMCELL** | 18.63 | 18.77 | 19.79 | 1.02 | 0.493116 | 0.628015 |
|  | 18.87 |  | 19.24 | 0.47 | 0.721965 |  |
|  | 18.81 |  | 19.35 | 0.58 | 0.668964 |  |
| **Aggrewell^TM^400** | 23.03 | 22.94333 | 22.57 | -0.37333 | 1.295342 | 1.422464 |
|  | 22.92 |  | 22.47 | -0.47333 | 1.388313 |  |
|  | 22.88 |  | 22.28 | -0.66333 | 1.583738 |  |
|  |  |  |  |  |  |  |
|  | **GAPDH (Ct)** | **AVG(GAPDH)** | **Desmin (Ct)** | **ΔCt Desmin** | **R** | **AVG (R)** |
| **2D** | 18.14 | 18.15 | 23.21 | 5.06 | 0.029977 | 0.031723 |
|  | 18.17 |  | 23.04 | 4.89 | 0.033726 |  |
|  | 18.14 |  | 23.14 | 4.99 | 0.031467 |  |
| **PAMCELL** | 18.63 | 18.77 | 23.41 | 4.64 | 0.040107 | 0.042508 |
|  | 18.87 |  | 23.07 | 4.3 | 0.050766 |  |
|  | 18.81 |  | 23.54 | 4.77 | 0.036651 |  |
| **Aggrewell^TM^400** | 23.03 | 22.94333 | 26.66 | 3.716667 | 0.076063 | 0.095856 |
|  | 22.92 |  | 26.22 | 3.276667 | 0.103187 |  |
|  | 22.88 |  | 26.15 | 3.206667 | 0.108317 |  |
|  |  |  |  |  |  |  |
|  | **GAPDH (Ct)** | **AVG(GAPDH)** | **Conexin 43 (Ct)** | **ΔCt Conexin 43** | **R** | **AVG (R)** |
| **2D** | 18.14 | 18.15 | 22.56 | 4.41 | 0.047039 | 0.045262 |
|  | 18.17 |  | 22.78 | 4.63 | 0.040386 |  |
|  | 18.14 |  | 22.52 | 4.37 | 0.048361 |  |
| **PAMCELL** | 18.63 | 18.77 | 20.5 | 1.73 | 0.301452 | 0.294913 |
|  | 18.87 |  | 20.41 | 1.64 | 0.320856 |  |
|  | 18.81 |  | 20.7 | 1.93 | 0.262429 |  |
| **Aggrewell^TM^400** | 23.03 | 22.94333 | 27.53 | 4.586667 | 0.041617 | 0.069782 |
|  | 22.92 |  | 26.57 | 3.626667 | 0.080959 |  |
|  | 22.88 |  | 26.47 | 3.526667 | 0.08677 |  |
|  |  |  |  |  |  |  |
|  | **GAPDH (Ct)** | **AVG(GAPDH)** | **cTnT (Ct)** | **ΔCt cTnT** | **R** | **AVG (R)** |
| **2D** | 18.14 | 18.15 | 21.71 | 3.56 | 0.084788 | 0.091055 |
|  | 18.17 |  | 21.49 | 3.34 | 0.098755 |  |
|  | 18.14 |  | 21.63 | 3.48 | 0.089622 |  |
| **PAMCELL** | 18.63 | 18.77 | 20.88 | 2.11 | 0.231647 | 0.242326 |
|  | 18.87 |  | 20.72 | 1.95 | 0.258816 |  |
|  | 18.81 |  | 20.85 | 2.08 | 0.236514 |  |
| **Aggrewell^TM^400** | 23.03 | 22.94333 | 24.33 | 1.386667 | 0.382447 | 0.376589 |
|  | 22.92 |  | 24.3 | 1.356667 | 0.390483 |  |
|  | 22.88 |  | 24.43 | 1.486667 | 0.356836 |  |

|  | **GAPDH (Ct)** | **AVG(GAPDH)** | **CACNA1C (Ct)** | **ΔCt CACNA1C** | **R** | **AVG (R)** |
| --- | --- | --- | --- | --- | --- | --- |
| **2D** | 18.63 | 18.77 | 24.97 | 6.2 | 0.013602 | 0.013 |
|  | 18.87 |  | 25.12 | 6.35 | 0.012259 |  |
|  | 18.81 |  | 25.02 | 6.25 | 0.013139 |  |
| **PAMCELL** | 18.14 | 18.15 | 24.55 | 6.4 | 0.011842 | 0.012297 |
|  | 18.17 |  | 24.52 | 6.37 | 0.01209 |  |
|  | 18.14 |  | 24.42 | 6.27 | 0.012958 |  |
| **Aggrewell^TM^400** | 23.03 | 22.94333 | 27.47 | 4.526667 | 0.043385 | 0.038212 |
|  | 22.92 |  | 27.53 | 4.586667 | 0.041617 |  |
|  | 22.88 |  | 28.02 | 5.076667 | 0.029633 |  |

|  | **GAPDH (Ct)** | **AVG(GAPDH)** | **KCNJ3 (Ct)** | **ΔCt KCNJ3** | **R** | **AVG (R)** |
| --- | --- | --- | --- | --- | --- | --- |
| **2D** | 19.15 | 19.10667 | 28.49 | 9.383333 | 0.001497 | 0.000909 |
|  | 19.05 |  | 29.85 | 10.74333 | 0.000583 |  |
|  | 19.12 |  | 29.7 | 10.59333 | 0.000647 |  |
| **PAMCELL** | 19.57 | 19.55 | 28.71 | 9.16 | 0.001748 | 0.001885 |
|  | 19.65 |  | 28.73 | 9.18 | 0.001724 |  |
|  | 19.43 |  | 28.39 | 8.84 | 0.002182 |  |
| **Aggrewell^TM^400** | 23.03 | 22.94333 | 30.12 | 7.176667 | 0.006912 | 0.006681 |
|  | 22.92 |  | 30.15 | 7.206667 | 0.00677 |  |
|  | 22.88 |  | 30.24 | 7.296667 | 0.00636 |  |
|  |  |  |  |  |  |  |
|  | **GAPDH (Ct)** | **AVG(GAPDH)** | **SCN5A (Ct)** | **ΔCt SCN5A** | **R** | **AVG (R)** |
| **2D** | 19.15 | 19.10667 | 25.86 | 6.753333 | 0.009269 | 0.008122 |
|  | 19.05 |  | 25.97 | 6.863333 | 0.008589 |  |
|  | 19.12 |  | 26.37 | 7.263333 | 0.006509 |  |
| **PAMCELL** | 19.57 | 19.55 | 24.8 | 5.25 | 0.026278 | 0.026806 |
|  | 19.65 |  | 24.85 | 5.3 | 0.025383 |  |
|  | 19.43 |  | 24.67 | 5.12 | 0.028756 |  |
| **Aggrewell^TM^400** | 23.03 | 22.94333 | 28.28 | 5.336667 | 0.024746 | 0.0245 |
|  | 22.92 |  | 27.88 | 4.936667 | 0.032652 |  |
|  | 22.88 |  | 28.9 | 5.956667 | 0.016101 |  |

**Supplementary Table S4.** Size distribution of 3D iPSC-cardiomyocyte spheroids grown on Aggrewell and PAMCELL plate.

| **Number** | **Spheroid diameter (μm)** | |
| --- | --- | --- |
|  | **Aggrewell** | **PAMCELL** |
| 1 | 101.45 | 89.37 |
| 2 | 107.4 | 91.35 |
| 3 | 95.61 | 99.97 |
| 4 | 103.63 | 97.64 |
| 5 | 92.62 | 85.99 |
| 6 | 101.12 | 80.18 |
| 7 | 83.07 | 93.2 |
| 8 | 83.66 | 91.96 |
| 9 | 93.86 | 95.37 |
| 10 | 89.83 | 102.65 |
| 11 | 90.21 | 90.5 |
| 12 | 89.53 | 83.89 |
| 13 | 86.2 | 69.19 |
| 14 | 104.39 | 104.24 |
| 15 | 90.15 | 67.45 |
| 16 | 88.45 | 90.88 |
| 17 | 106.6 | 76.748 |
| 18 | 85.06 | 80.664 |
| 19 | 96.53 | 81.447 |
| 20 | 95.36 | 76.748 |
| 21 | 100.43 | 59.519 |
| 22 | 92.86 | 83.797 |
| 23 | 84.11 | 97.893 |
| 24 | 88.45 | 117.472 |
| 25 | 89.21 | 100.026 |
| 26 | 82.72 | 77.532 |
| 27 | 96.35 | 90.845 |
| 28 | 96.42 | 97.893 |
| 29 | 83.87 | 103.38 |
| 30 | 94.48 | 112.07 |
| 31 | 90.18 | 101.71 |
| 32 | 73.91 | 121.08 |
| 33 | 88.13 | 92.25 |
| 34 | 111.74 | 97.79 |
| 35 | 88.41 | 95.69 |
| 36 | 90.76 | 85.65 |
| 37 | 92.8 | 90.52 |
| 38 | 85.56 | 81.03 |
| 39 | 82.66 | 84.89 |
| 40 | 96.33 | 110.65 |
| 41 | 77.3 | 106.34 |
| 42 | 94.21 | 119.84 |
| 43 | 91.32 | 80.37 |
| 44 | 103.55 | 106.04 |
| 45 | 98.93 | 72.5 |
| 46 | 98.38 | 85.36 |
| 47 | 89.16 | 101.9 |
| 48 | 92.93 | 88.44 |
| 49 | 94.28 | 87.09 |
| 50 | 91.21 | 104.25 |

**Supplementary Table S5.** Beating period of iPSC-cardiomyocytes on 2D platform and, Aggrewell and PAMCELL plate.

| **Number** | **Beating period (ms)** | | |
| --- | --- | --- | --- |
|  | **2D** | **Aggrewell** | **PAMCELL** |
| 1 | 1737.5 | 1344.444 | 922.857 |
| 2 | 1728.571 | 1346.667 | 1185 |
| 3 | 1725 | 1357.143 | 1070 |
| 4 | 1716.667 | 1453.333 | 1066.67 |
| 5 | 1733.333 | 1455.556 | 866.7 |
| 6 | 1725 | 1444.444 | 1012.903 |
| 7 | 1725 | 1554.235 | 852.381 |
| 8 | 1725 | 1464.581 | 918.18 |
| 9 | 1712.5 | 1642.578 | 945.45 |
| 10 | 1728.571 | 1462.145 | 1070 |
| 11 | 1722.222 | 1321.466 | 1070 |
| 12 | 1725 | 1657.976 | 1177.778 |
| 13 | 1725 | 1666.667 | 1163.056 |
| 14 | 1725 | 1321.333 | 1263.889 |
| 15 | 1725 | 1587.965 | 1165.385 |
| 16 | 1722.22 | 1565 | 1120 |
| 17 | 1725 | 1458.594 | 857.623 |
| 18 | 1725 | 1388.354 | 1025 |
| 19 | 1722.222 | 1553.667 | 1060 |
| 20 | 1725 | 1478.33 | 1070 |
